# High-performance polarization management devices based on thin-film lithium niobate

**DOI:** 10.1038/s41377-022-00779-8

**Published:** 2022-04-13

**Authors:** Zhongjin Lin, Yanmei Lin, Hao Li, Mengyue Xu, Mingbo He, Wei Ke, Heyun Tan, Ya Han, Zhaohui Li, Dawei Wang, X. Steve Yao, Songnian Fu, Siyuan Yu, Xinlun Cai

**Affiliations:** 1 State Key Laboratory of Optoelectronic Materials and Technologies, School of Electronics and Information Technology, Sun Yat-sen University, 510275 Guangzhou, China; 2 Department of Electrical and Computer Engineering, The University of British Columbia, Vancouver, BC V6T 1Z4 Canada; 3 Photonics Information Innovation Center and Hebei Provincial Center for Optical Sensing Innovations, College of Physics Science and Technology, Hebei University, 071002 Baoding, China; 4 Institute of Advanced Photonics Technology, School of Information Engineering, Guangdong University of Technology, 510006 Guangzhou, China

**Keywords:** Optoelectronic devices and components, Integrated optics, Photonic devices

## Abstract

High-speed polarization management is highly desirable for many applications, such as remote sensing, telecommunication, and medical diagnosis. However, most of the approaches for polarization management rely on bulky optical components that are slow to respond, cumbersome to use, and sometimes with high drive voltages. Here, we overcome these limitations by harnessing photonic integrated circuits based on thin-film lithium niobate platform. We successfully realize a portfolio of thin-film lithium niobate devices for essential polarization management functionalities, including arbitrary polarization generation, fast polarization measurement, polarization scrambling, and automatic polarization control. The present devices feature ultra-fast control speeds, low drive voltages, low optical losses and compact footprints. Using these devices, we achieve high fidelity polarization generation with a polarization extinction ratio up to 41.9 dB and fast polarization scrambling with a scrambling rate up to 65 Mrad s^−1^, both of which are best results in integrated optics. We also demonstrate the endless polarization state tracking operation in our devices. The demonstrated devices unlock a drastically new level of performance and scales in polarization management devices, leading to a paradigm shift in polarization management.

## Abstract (web summary)

This work successfully demonstrates that thin-film lithium niobate platform can bring polarization management devices into a new phase of that higher speed, smaller size, and lower cost.

## Graphical abstract

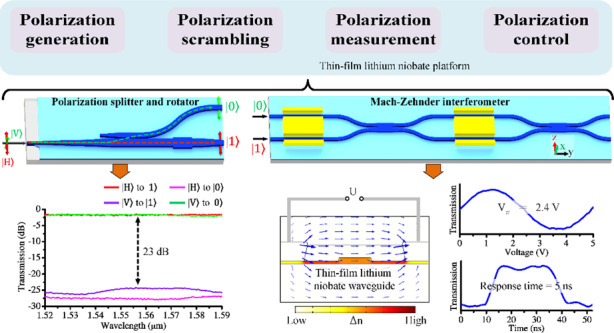

## Introduction

State of polarization (SOP), the vectorial signature of light, is of paramount importance for both fundamental research and practical applications. Unlike insects and some vertebrates that possess sensory mechanisms to perceive polarized light patterns for navigation^1–3^, humans rely on specific devices to control and use the SOP of light for widespread applications, including remote sensing^4^, telecommunication^5^, medical diagnosis^6^, and material analysis^7^. Although the polarization management device has been studied for several hundred years, up to date, it still attracts lots of attentions, especially after introducing micro-nano technology^8,9^. Some new applications of the polarization management device are continuously developing^10^.

Polarization management devices capable of fast and dynamic control over the SOP are highly desirable. For example, by analyzing the SOP of optical signal, light remote detection and ranging (LiDAR) systems can reveal the profiles and types of aerosol particles, which is important for monitoring air pollution and predicting climate change^11^. In this case, devices for SOP generation and measurement, with high-speed and high-accuracy, are crucial for improving the throughput and spatial-temporal resolutions of SOP LiDAR systems. In optical fiber communication systems, an automatic polarization control device can be used to track and stabilize the fluctuation of SOPs at the receiver end^12,13^. This approach has the potential to simplify digital signal processing algorithms and reduce the power consumption. In this scenario, the high-speed devices with an unlimited transformation range, or “endless” operation, are required to avoid any interruptions or reset processes.

To date, most of the polarization management devices are based on mechanically rotated wave-plates or fiber-optic coils^14,15^, but these solutions are slow and may introduce instabilities from mechanical vibrations. Higher speed can be achieved with liquid crystal devices^16–18^, combining fibers with piezo-electric actuators^19^, or combining waveplates with magneto-optic crystals^20^. However, all these approaches rely on bulk-optic components and the control speed is limited to the milli- or micro-second levels. Electro-optic (EO) polarization management in Ti-indiffused lithium niobate (LN) can obtain very fast control speeds on the order of nanoseconds, where the electrical field applied to the Ti-indiffused LN waveguide enables modification of SOP through the Pockels effect^12,13^. While Ti-indiffused LN devices are attractive for fast polarization management, the performance of these devices is already reaching the physical limits that the Ti-indiffused LN waveguides can ever support. The current off-the-shelf LN polarization management device is still bulky in size (larger than 5 cm) and suffer from a high half-wave voltage (larger than 10 V)^13,21^, which severely limit their applications in communications and sensing.

Recently, thin-film LN (TFLN) has emerged as a promising platform for future EO integrated devices^22–26^. This platform seamlessly combines the superior EO modulation property of LN material with high-index-contrast waveguide structure. As a result, the TFLN devices exhibit much lower drive voltages and much smaller size, compared to their conventional counterparts^27^. Here, we report a portfolio of TFLN-based photonic integrated circuits (PICs) capable of realizing the essential polarization management functionalities, including the arbitrary polarization generation, fast polarization scrambling, fast polarization measurement, and endless automatic polarization control. These devices feature ultra-fast control speeds, low drive voltages, low optical losses and compact footprints, many of which exhibit performance well beyond the state-of-the-art.

## Results

### Basic building blocks

**Fig. 1:**
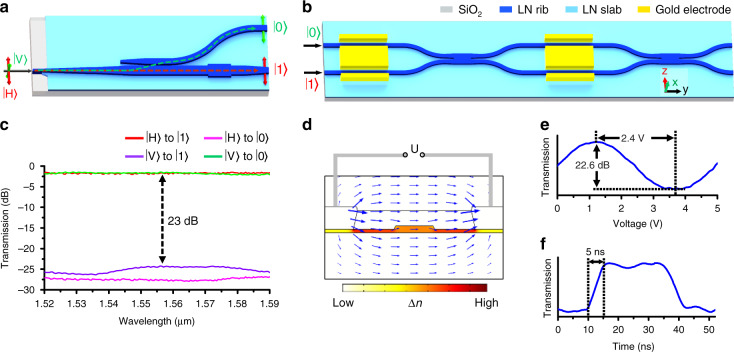
Architecture and performance of the fundamental building blocks. **a** The schematics of the polarization splitter and rotator (PSR). $\left| {{{\mathrm{V}}}} \right\rangle$ and $\left| {{{\mathrm{H}}}} \right\rangle$ represent the vertically and horizontally polarized components of light in free space, respectively. $\left| 0 \right\rangle$ and $\left| 1 \right\rangle$ represent fundamental TE modes of upper and lower waveguides, respectively. **b** The architecture of 2 × 2 Mach-Zehnder interferometer (MZI), composed of two electro-optic (EO) phase shifters. **c** The measured transmissions of the fabricated PSR (including the off-chip coupling loss) when injecting horizontal polarization $\left| {{{\mathrm{H}}}} \right\rangle$ and vertical polarization $\left| {{{\mathrm{V}}}} \right\rangle$, respectively. **d** The numerical result of the refractive index variation distribution of the lithium niobate (LN) waveguide when applying the voltage to the electrode. **e** The normalized transmission of a MZI as a function of an applied voltage, showing a half-wave voltage of 2.4 V and an extinction ratio of 22.6 dB. **f** The normalized transmission of a MZI as a function of time when applying rectangle wave signals, indicating a switching speed of <5 ns

In this work, all the demonstrated TFLN PICs are based on two fundamental building blocks. The first one is the polarization splitter and rotator (PSR)^28–30^, which maps the orthogonal linearly polarized states in free space, $\left| {{{\mathrm{V}}}} \right\rangle$ and $\left| {{{\mathrm{H}}}} \right\rangle$, into two guided modes in two TFLN waveguides, $\left| 0 \right\rangle$ and $\left| 1 \right\rangle$, and vice versa.$\left| 0 \right\rangle$ and $\left| 1 \right\rangle$ represent fundamental TE modes of upper and lower waveguides in Fig. 1a, respectively. The second one is a 2 × 2 Mach-Zehnder interferometer (MZI), composed of two voltage-controlled EO phase shifters and two 3-dB multi-mode interferometer (MMI) couplers (Fig. 1b). The relative amplitude and phase differences between $\left| 0 \right\rangle$ and $\left| 1 \right\rangle$ can be reconfigured by the first and second EO phase shifter. In general, this architecture allows arbitrary unitary transformation to be performed within the PICs, which in turn manipulate the SOP in the output port.

We designed and fabricated the two building blocks on an X-cut TFLN platform, both of which exhibit high performance. The device design and the fabrication process are detailed in Supplementary Material I–IV and XI. The principle of the PSR is based on a mode evolution scheme, which consists of a mode evolution taper, an asymmetric directional coupler, and an edge coupler for off-chip coupling. The PSR features a polarization cross talk of near 23 dB, an operation bandwidth of larger than 70 nm, and an on-chip insertion loss of lower than 0.12 dB (see Fig. 1c). Moreover, the device also exhibits an off-chip coupling loss of near 1.7 dB for both polarizations with a polarization-dependent loss (PDL) of 0.15 dB. The MZI operates in a single-drive push–pull configuration, so that the electric fields induce phase shifts (see Fig. 1d) with an equal magnitude but opposite sign in the two arms. A critical figure of merit for MZI is the half-wave voltage (*V*_π_), defined as the voltage required to switch the transmission from cross to bar states. For the MZI used in the present devices, the arm length is 1.2 cm. We measure a *V*_π_ of 2.4 V (see Fig. 1e) and a switching speed of near 5 ns (see Fig. 1f), which facilitates high-speed and low-power operation. Importantly, the MZI also features a low insertion loss of lower than 0.4 dB and an extinction ratio (ER) of near 22.6 dB.

### Arbitrary SOP generation

**Fig. 2:**
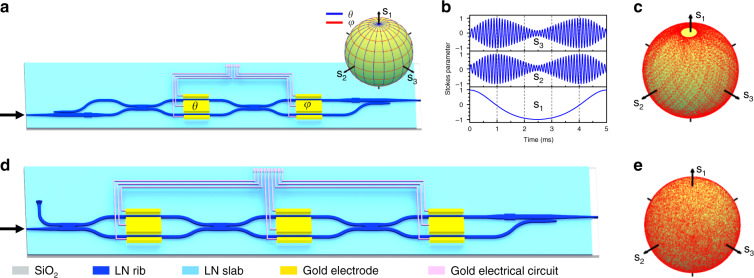
Performance of the device for arbitrary SOP generation. **a** The schematics of the device for the arbitrary SOP generation. Inset: the position of SOP on the Poincaré sphere when changing *θ* (blue line) or *φ* (red line) of the electro-optic (EO) phase shifters. **b** The measured Stokes vectors as a function of time when applying two triangle wave signals with frequencies of 200 Hz and 10 kHz to *θ* and *φ* EO phase shifter, respectively. **c** The measured Stokes vectors on the Poincaré sphere of the output from the device, indicating a polarization extinction ratio (PER) of 22.8 dB. **d** The schematics of a multi-stage device with an improved PER. **e** The measured Stokes vectors of the output from the multi-stage device, indicating a PER of 41.9 dB

Arbitrary SOP generation can be implemented using two PSRs and one MZI, as illustrated in Fig. 2a. We set *θ* and φ as the phase difference induced by the first and second EO phase shifters, respectively. When the input SOP is set at $\left| {{{\mathrm{H}}}} \right\rangle$, the normalized Stokes vector **S** of the output SOP can be expressed by

1 $${{{\mathbf{S}}}} = (s_0\,s_1\,s_2\,s_3)^T = (1\,\cos\theta\, -\sin\theta\cos \varphi\, -\sin\theta\sin\varphi )^T$$

where *s*_0_, *s*_1_, *s*_2_, and *s*_3_ are the four Stokes parameters. The north and south poles of the Poincaré sphere are represented by the points of *s*_1_ = 1 and *s*_1_ = −1, respectively. The derivation of Eq. (1) is in Supplementary Material V. Equation (1) indicates that we can independently control the longitude or latitude position of the output SOP on the Poincaré sphere by *θ* or *φ* (inset of Fig. 2a).

We test our arbitrary SOP generating device by applying two triangle wave signals with frequencies of 200 Hz and 10 kHz to *θ* and *φ* phase shifters, respectively. The peak-to-peak drive voltages (*V*_pp_) are set to be 4.8 V, corresponding to 2π phase shift. In this case, the device is supposed to sample over the entire Poincaré sphere. We use a commercial polarization analyzer (General Photonics PSY 201) to characterize the generated SOP from our device. The waveform of the modulated stokes parameters and the sampling points on the Poincaré sphere are depicted in Fig. 2b, c, respectively. The repeatability of generated SOPs at given voltages are provided in Supplementary Material V. The polarization extinction ratio (PER) was measured to be 22.8 dB. As expected, the PER of the device coincides with the ER of MZI. We note that the limited PER leads to two un-sampled areas in the vicinity of the north and south poles in Fig. 2c. In principle, the higher PER can be achieved by improving the ER of MZI. However, the ER of MZI is difficult to improve in practice because the fabrication imperfections that can cause the splitting ratio of MMI to deviate from 50:50 always exist.

To fully overcome the restriction of limited ER in MZI, we further demonstrate a multi-stage device, in which additional interferometers function^31^ as beam splitters with variable splitting ratio (Fig. 2d). The Poincaré sphere can be fully covered by the SOP generated from this device (Fig. 2e). The measured PER is 41.9 dB, which is the best value in integrated optics^32^. The details of this multi-stage device can be found in Supplementary Material V.

### Polarization scrambling

**Fig. 3:**
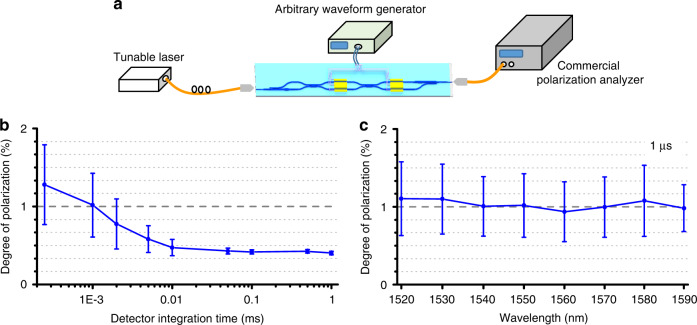
Performance of the polarization scrambling device. **a** The experiment setup for characterizing the performance of the polarization scrambling device. **b** The degree of polarization (DOP) as a function of the detector integration time at a wavelength of 1550 nm. **c** The DOP as a function of the wavelength when the detector integration time is equal to 1 μs

We programmed our arbitrary SOP generating device to implement fast polarization scrambling (Fig. 3a), which can be used to mitigate polarization related impairments in optical fiber communication and sensing systems. An important figure of merit for polarization scrambling device is the scrambling rate, defined as the rate of polarization change on the surface of Poincaré sphere. The scrambling rate needs to be fast enough so that the average polarization over certain period of time effectively covers the entire surface of Poincaré sphere. Figure 3a depicts the experimental setup for testing the scrambling rate. We applied two triangle waves with frequencies of 4.242 MHz and 6 MHz to *θ* and *φ* phase shifters, respectively. In this case, the corresponding scrambling rate is 65 Mrad s^−1^ (see Eq. (S18) of Supplementary Material VII). A commercial polarization analyzer was used to measure the degree of polarization (DOP) of the output from our device. Figure 3b presents the DOP as a function of the detector integration time at a wavelength of 1550 nm. The result indicated that a DOP of 1 % can be achieved at a detector integration time of only 1 μs. The DOP as a function of the scrambling rate is provided in Supplementary Material VII. Figure 3c shows the measured DOP at different wavelength for a detector integration time of 1 μs, indicating broadband operation of the device over 70 nm. These results demonstrate that our scrambling device significantly outperforms the best commercial scrambling device (http://139.224.26.190/storage//file//data//tmp//180118032700.pdf) with a maximum scrambling rate of 50 Mrad s^−1^.

### Fast polarization measurement

**Fig. 4:**
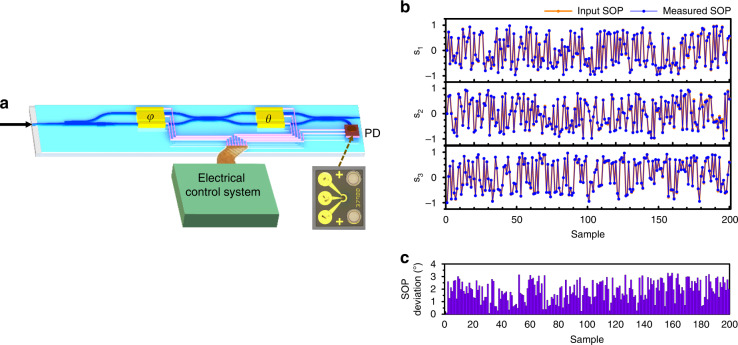
Performance of the device for fast polarization measurement. **a** The experiment setup used for characterizing the fast polarization measuring device. Inset: the microscope image of a flip-chip bonded photodetector (PD). According to the optical intensities recorded by the PD, the input state of polarization (SOP) can be reconstructed. **b** The measured results (blue line) from the device and the corresponding input polarization states (orange line). **c** SOP deviation between the input SOP and the measured one

We realized fast and accurate SOP measurement using a TFLN PIC composed of a PSR, a MZI and a flip-chip bonded photodetector (PD), as depicted in Fig. 4a. It has an architecture similar to the SOP generating device, but with the PD for monitoring the optical intensity. In general, the SOP can be measured by making four projective intensity measurements onto four sets of predetermined measurement basis^33–35^ which are parameterized by a 4 × 4 analysis matrix, **W**. The Stocks vector is then given by **S** = **W**^−1^**I**, where **I** = (*I*_1_, *I*_2_, *I*_3_, *I*_4_) is a 4-dimensional vector representing the results of projective intensity measurements. In our case, *I*_1_, *I*_2_, *I*_3_, and *I*_4_ are measured by the PD and the matrix elements of **W** are determined by setting different values on *θ* and *φ*.

We adopted the “optimal frames” protocol, which has been developed as an effective method to minimize the influence of the noise (see Supplementary Material VI)^33^, to perform fast SOP measurement (Fig. 4a). An input optical signal with rapidly and randomly varying SOP, generated from a commercial polarization synthesizer/analyzer, was used to interrogate the performance of our device. Figure 4b shows the comparison of the measured SOP from our device and the original input SOP, both of which are parameterized by Stokes vector elements. *s*_0_ is normalized to 1. Figure 4c presents the angle deviation between the input SOP and the measured one. How to calculate the angle deviation can be found in Supplementary Material VI. The results from our device exhibited high level of accuracy with the RMS deviation of 1.58°. Moreover, the sampling rate of the device can go up to 250 KS/s (detail can be found in Supplementary Material VI) without compromising the detection accuracy, thanks to the ultra-fast response of the TFLN material. Our device can also be used to measure the DOP of the input light beam with an accuracy of 2.5%, which is detailed in Supplementary Material VI.

### Endless automatic polarization control

An essential requirement of automatic polarization control is that it must be able to operate in an endless way. In other words, the polarization control device is capable of continuously tracking the rapidly varying changes of all possible SOPs, even if the SOPs wander infinite times around the Poincaré sphere^13^. The PIC with one PSR and one MZI which includes two EO phase shifters is sufficient for transforming any input SOP to the TE mode, but as the input SOP evolves, the drive voltages could reach the boundaries and a reset process is then required (see Supplementary Material VIII), resulting in momentary SOP mismatch and data interruption. Here we adopted a multi-stage interferometers with four electrodes to facilitate an endless and seamless operation^36^. By controlling the applied voltages of the four electrodes within 4 *V*_π_, the proposed device can endlessly track the input SOP.

**Fig. 5:**
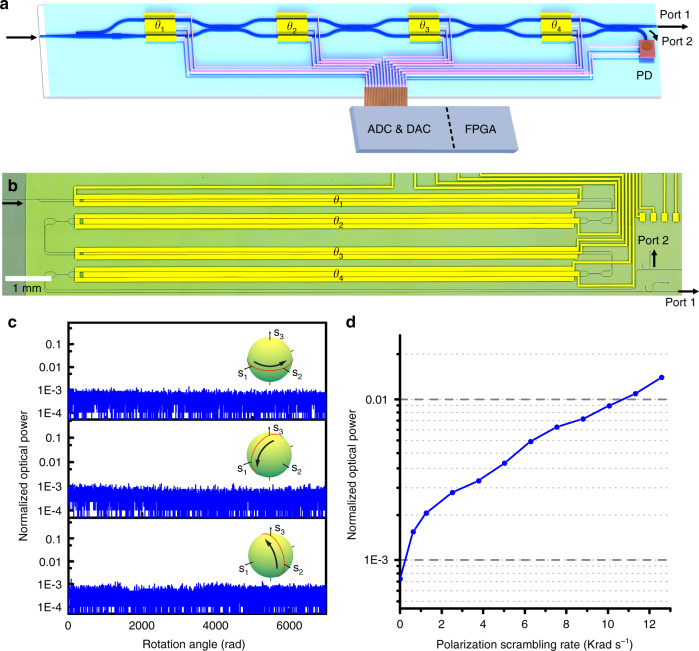
The performance of the endless automatic polarization controlling device. **a** The schematics of the proposed endless automatic polarization controlling device. DAC digital-to-analog converter, ADC analog-to-digital converter, FPGA field-programmable gate array, PD flip-chip bonded photodetector. **b** The microscope image of the fabricated device. *θ*_1_, *θ*_2_, *θ*_3_ and *θ*_4_ in **a** and **b** mark the phase shifters. **c** The normalized optical power of the PD as a function of the rotation angle of the input polarization. Insets: the rotation trajectories of the input polarization states for demonstrating the capability of the endless operation. **d** The normalized optical power of the feedback signal as a function of the polarization scrambling rate

We developed the TFLN endless automatic polarization control device, composed of a PSR, a flip-chip bonded PD, and a multi-stage MZI with four EO phase shifters, as depicted in Fig. 5a. The PD was used to monitor the optical power from one of the output waveguide (port 2 in Fig. 5a), which was further used as a feedback to control the voltages applied onto the four EO phase shifters. A gradient algorithm was digitally implemented in a field-programmable gate array (FPGA) for fast execution. The algorithm was designed to minimize the power received by the PD under all possible input SOPs. Thus all of the power of the input signal with arbitrary SOP would be transferred to the other output waveguide with TE polarization (port 1 in Fig. 5a). Figure 5b depicted the micrograph of the fabricated devices. The device is folded to reduce the total length, and the footprint is ~1.5 cm × 0.3 cm. We measured an on-chip insertion loss of 0.92 dB for the device.

We use a commercial polarization scrambling device to test the performance of our polarization control device. Two steps are taken to confirm that our device can operate in an endless way and achieve a high tracking speed. Firstly, to verify the endless operation capability of our device, the input SOPs were programmed to endlessly rotate around three different orthodromes on the Poincaré sphere at the speed of 12.56 rad s^−1^ (Fig. 5c insets). We perform tracking experiment for more than 10 min for each great orthodrome, corresponding to a SOP change of more than 7000 rad. Figure 5c shows that the normalized optical power received by the PD was always kept at a low level of lower than 0.1%, indicating the successful endless tracking operation. To further test the tracking rate of our device, we reprogrammed the commercial polarization scrambler to generate rapidly and randomly varying SOPs at different scrambling rate, and then we used our device to track and stabilize the scrambled SOPs. As shown in Fig. 5d, the normalized power received by the flip-chip bonded PD could still be maintained at around 1%, even at the SOP changing rate of 10 Krad s^−1^. This is by far the highest endless tracking rate reported in integrated photonics. It should be noted that the faster tracking rate can be achieved by simply upgrading the electronic control system.

**Fig. 6:**
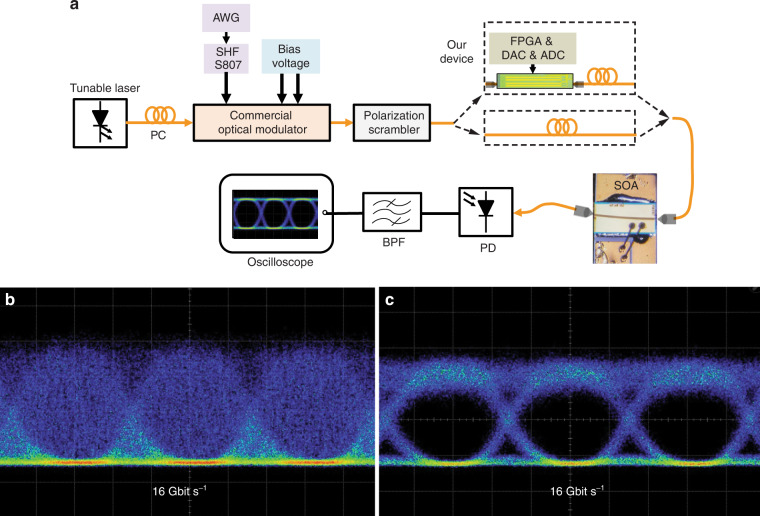
The experiment for demonstrating the application in the communication system. **a** A schematic of the experiment setup. PC polarization controller based on the coiling fiber, AWG arbitrary waveform generator, FPGA field programmable gate array, SOA semiconductor optical amplifier, PD photodetector, BPF band pass filter, DAC digital-to-analog converter, ADC analog-to-digital converter. **b** and **c** are eye diagrams in the cases of the systems including and excluding our on-chip endless automatic polarization controlling device, respectively

To show an application in the communication, we combine our polarization controlling device with a semiconductor optical amplifier (SOA) to mitigate the polarization-dependent gain of SOA which can be used to improve the sensitivity of the optical receiver. Figure 6a presents a schematic of the experiment setup. More details about the experiment are provided in Supplementary Material XII. The SOP of light emitted from a transmitter was scrambled at a variation rate of 6.28 Krad s^−1^. Figure 6b, c show the eye diagrams when the system is without and with our device, respectively.

## Discussion

We have successfully demonstrated high-performance TFLN-based PICs promising for polarization management applications. The TFLN material provides a stable, compact and robust platform to implement high-speed and exquisite polarization management. Sophisticated devices can be fabricated in a mass-produced way by standard semiconductor process, such as lithography, etching and metal patterning. Indeed, this approach brings new levels of performance, functionality, and scalability to polarization management.

It should be noted that polarization management functionalities have also been implemented on other material platform, such as silicon-on-insulator (SOI)^29,32,37–40^, indium phosphide (InP)^41,42^, plasmonics^43^, and conventional LN^21^. In Table 1, we compare the performance metrics of our device to the state-of-the-art. Clearly, the present device features the fastest response speed and lowest drive voltage. To the best of our knowledge, these are also the records for all polarization devices. In particular, the low drive voltage of our device is highly attractive for high-speed and power-efficient operation. The low optical loss of our device is also very appealing. For example, the endless polarization control device features an on-chip insertion loss of only 0.92 dB. Therefore, it can be monolithically integrated with other TFLN devices and balance PDs to form integrated coherent receivers capable of polarization de-multiplexing, bringing new possibilities to future high bandwidth and low power consumption optical networks. Importantly, the TFLN platform provides pure phase modulation which means the intensity of the light does not change with an external modulation voltage. This fundamentally avoids the activation loss commonly observed in silicon and InP devices. In fact, the measured activation loss in our device is negligible (see Supplementary Material V). Furthermore, TFLN platform support a wide transparent window from 400 nm to 5000 nm^44^, compared to other material systems. This also opens new application opportunities in areas like biology, chemistry, medicine, remote-sensing, and astronomy. We have summarized some potential applications in Supplementary Material XIII. In addition to the application in communication, in this work, we also experimentally demonstrated that our device can be used to develop a body joint motion sensor which is important for studying musculoskeletal disorders, studying the behavior of the animals, and improving the action of the athletes (Supplementary Material XIV).

**Table 1:** The comparison of several performance metrics of the active integrated polarization management devices

Platform	Principle	*V*_π_ (V)	Length (cm)	Response time (ns)	Optical losses^a^ (dB)	Transparent window (μm)
SOI^37,45,46^	Thermo-optics effect	<10	NA	>5 × 10^4^	NA	1–5^47^
SOI^48^	Plasma dispersion effect	7.07	NA	<2.5	5	1–5^47^
InP^41^	Plasma dispersion effect	<3	NA	NA	5.5	1.1–1.6^49^
Plasmonic^43^	Faraday effect	NA	NA	<1000	2.5^b^	NA
Ti:LiNbO_3_^21^	Pockels effect	~10	>5	<10	NA	0.4–5^44^
This work	Pockels effect	~2.4	1.5^c^	<5	0.52^d^ 0.92^e^	0.4–5^44^

*NA* not available.

^a^On-chip insertion loss.

^b^Only generating an arbitrary linear polarization state.

^c^is easy to be a smaller value.

^d^Including a PSR, and a single-stage MZI with two EO phase shifters.

^e^Including a PSR, and a multi-stage MZI with four EO phase shifters.

## Materials and methods

### Photonic chip fabrication

The devices were fabricated on a commercial X-cut LN-on-insulator wafer from NANOLN. The thicknesses of the LN and buried oxide layers are 360 nm and 4.7 µm, respectively. The fabrication process of the optical component is detailed in the following: electron beam lithography (EBL) was first used to define the rib waveguide structures on the hydrogen silses quioxane (HSQ) resist. Secondly, the patterns were transferred to the top LN layer with an etching depth of 180 nm by inductively coupled plasma (ICP) dry etching. Then, the strip waveguides for the edge coupler were defined on the LN layer with an etching depth of 360 nm using EBL and ICP dry etching. At last, a SiO_2_ layer with a thickness of 1 µm was deposited on the wafer as the upper-cladding by plasma-enhanced chemical vapor deposition (PECVD). More details can be found in Supplementary Material XI.

### Experiment setup

A commercial polarization synthesizer/analyzer (General Photonics, PSY 201) was used to perform the polarization measurement, polarization scrambling, and polarization generation for characterizing our devices. The commercial FPGA (NI, cRIO-9047), digital-to-analog converters (NI9262) and analog-to-digital converters (NI9223) were used to control our electrical system. Light from a tunable laser (Santec TSL-550) was injected into our chip. A commercial SOA (Thorlabs, BOA1007C) was used in the experiment for demonstrating the communication application. More details about characterizing our devices can be found Supplementary Material V–VIII.

## Supplementary information

Supplemental material

## Data Availability

All the data supporting the findings in this study are available in the paper and Supplementary Information. Additional data related to this paper are available from the corresponding authors upon request
