# Supplementary material for: High-performance polarization management devices based on thin-film lithium niobate
Source: Light Sci Appl. 2022 Apr 13;11:93. doi: 10.1038/s41377-022-00779-8 (PMC9008021; doi:10.1038/s41377-022-00779-8)
Supplement: Supplementary file 1 — Supplemental material [file 41377_2022_779_MOESM1_ESM.docx]

**Supporting information for “High Performance Polarization Management Devices Based on Thin-Film Lithium Niobate”**

Zhongjin Lin^1, 2, #^, Yanmei Lin^1, #^, Hao Li^1, #^, Mengyue Xu^1^, Mingbo He^1^, Wei Ke^1^, Heyun Tan^1^, Ya Han^1^, Zhaohui Li^1^, Dawei Wang^1^, X.Steve Yao^3^, Songnian Fu^4^, Siyuan Yu^1^, Xinlun Cai^1,^ *

1 State Key Laboratory of Optoelectronic Materials and Technologies, School of Electronics and Information Technology, Sun Yat-sen University, Guangzhou 510275, China

2 Department of Electrical and Computer Engineering, The University of British Columbia, Vancouver, BC V6T 1Z4, Canada

3 Photonics Information Innovation Center and Hebei Provincial Center for Optical Sensing Innovations, College of Physics Science and Technology, Hebei University, Baoding 071002, China

4 Institute of Advanced Photonics Technology, School of Information Engineering, Guangdong University of Technology, Guangzhou, 510006, China

# These authors contributed equally

* Corresponding author: [caixlun5@mail.sysu.edu.cn](mailto:caixlun5@mail.sysu.edu.cn)

Content

[I. The performance of the edge coupler 2](#_Toc92046683)

[II. The design of the polarization splitter and rotator 2](#_Toc92046684)

[III. The propagation losses of waveguide 4](#_Toc92046685)

[IV. The optimization of the gold electrodes 4](#_Toc92046686)

[V. The principle and experiment of the arbitrary polarization generation 4](#_Toc92046687)

[VI. The principle and experiment of the polarization measurement 7](#_Toc92046688)

[VII. The experiment for polarization scrambling 9](#_Toc92046689)

[VIII. The experiment for demonstrating polarization controlling device 10](#_Toc92046690)

[X. The summary of active bulky polarization management devices 12](#_Toc92046691)

[XI. Fabrication process 13](#_Toc92046692)

[XII. Application in communication 14](#_Toc92046693)

[XIII. Potential applications in the sensing fields 16](#_Toc92046694)

[XIV. Body joint motion sensor 18](#_Toc92046695)

[Reference 20](#_Toc92046696)

# I. The performance of the edge coupler

The schematic of the proposed polarization-independent edge coupler is presented in Fig. S1a. Both rib and slab portions of the waveguide utilize the architecture of gradual tapering. The top (rib) taper evolves from a width of 800 nm down to 80 nm with a height h_rib_ of 180 nm over a 150 μm length. The bottom (slab) layer taper narrows down laterally from 6 μm to a tip of 80 nm wide with a slab height h_slab_ of 180 nm. At the end, a strip waveguide with a width of 80 nm is used to match the optical mode of fiber.

The microscope and scanning electron images of the fabricated edge coupler are shown in the inset of Fig. S1b. At the wavelength of 1550 nm, the off-chip coupling losses of the edge coupler are 1.7 dB and 1.55 dB when injecting horizontal polarization and vertical polarization, respectively (Fig. S1), indicating a low polarization-dependent loss of 0.15 dB. The 3-dB bandwidth covers the range of 70 nm.


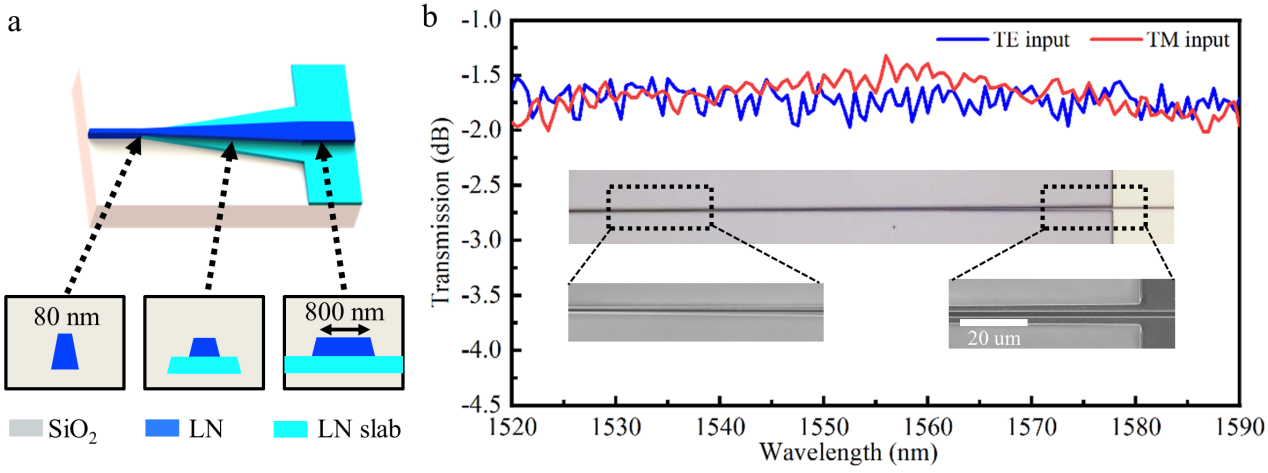


Fig. S1 **The performance of the polarization-independent edge coupler.** **a** The schematic of the proposed edge coupler. Insets: the profiles of different sections. **b** The measured transmissions of the fabricated edge coupler when injecting horizontal polarization (TE, blue line) and vertical polarization (TM, red line), respectively. Top of inset: the microscope image of the fabricated edge coupler. Bottom of inset: the scanning electron image of the special section of the fabricated edge coupler.

# II. The design of the polarization splitter and rotator

The schematic of the polarization splitter and rotator (PSR) is presented in Fig. S2a. It includes an adiabatic taper (AT), an asymmetrical directional coupler (ADC), and a multimode interferometer mode filter (MMIF). The AT is split into three sections. The widths of the three sections are 0.8 μm, 1.1 μm, 1.7 μm, and 2.16 μm, respectively. The corresponding lengths are 50 μm, 250 μm, and 50 μm, respectively. The length of the coupled region of the ADC is 34 μm. According to the phase-matching condition, the widths for the straight bus and access waveguides of the ADC are 2.16 μm and 0.8 μm, respectively. The width and length of the MMIF are 6 μm and 53.5 μm, respectively. Fig. S2b presents the microscope image of the fabricated PSR. We will introduce the principle of the PSR in the following.

The insets of Fig. S2b provide the mode evolution at different sections of the proposed device. When an arbitrary polarization state is injected into the AT from the right side, the TM_0_ mode is converted into the TE_1_ mode, but the launched TE_0_ mode does not undergo mode conversion. Then, the ADC is designed to split the TE_0_ and TE_1_ modes into the straight bus and access waveguides, respectively, and simultaneously converts the TE_1_ mode to the TE_0_ mode. The MMIF, connecting with the straight bus waveguide, is used for filtering out the residual TE_1_ mode so that a high extinction ratio can be achieved when the TM_0_ mode is launched. All the structures, including the AT section which is used for converting the TM mode to TE mode, are fabricated in a single lithography step, which indicates a high fabrication tolerance.


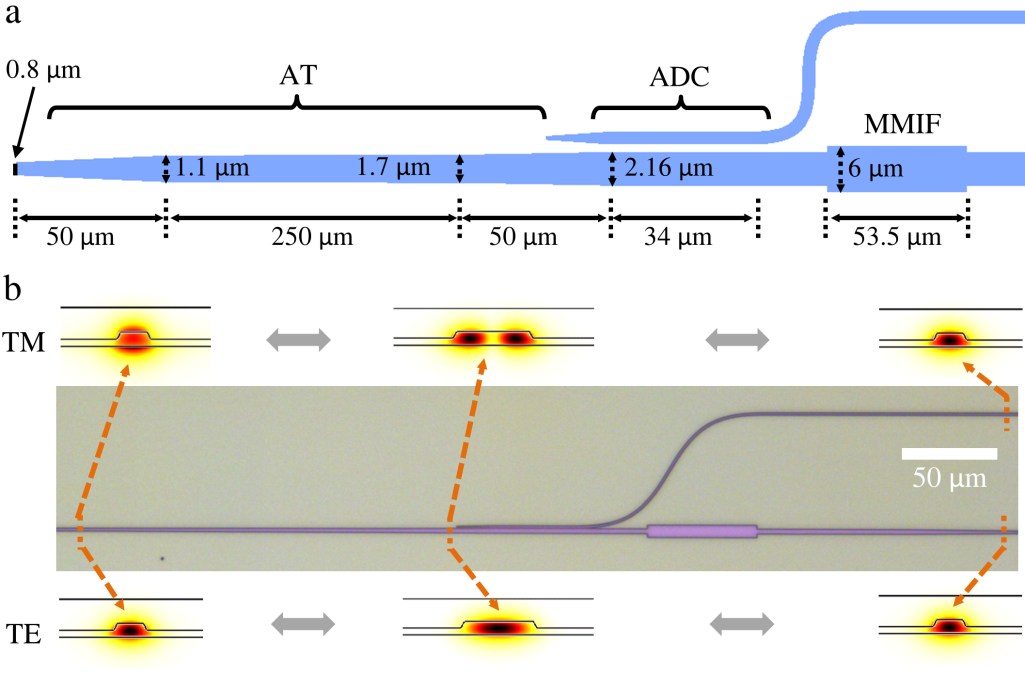


Fig. S2 **The schematic of the polarization splitter and rotator.** **a** The design of the polarization splitter and rotator. **b** The microscope image of the fabricated polarization splitter and rotator. Top: the evolution from TE to TE modes. Bottom: the evolution from TM to TE modes.


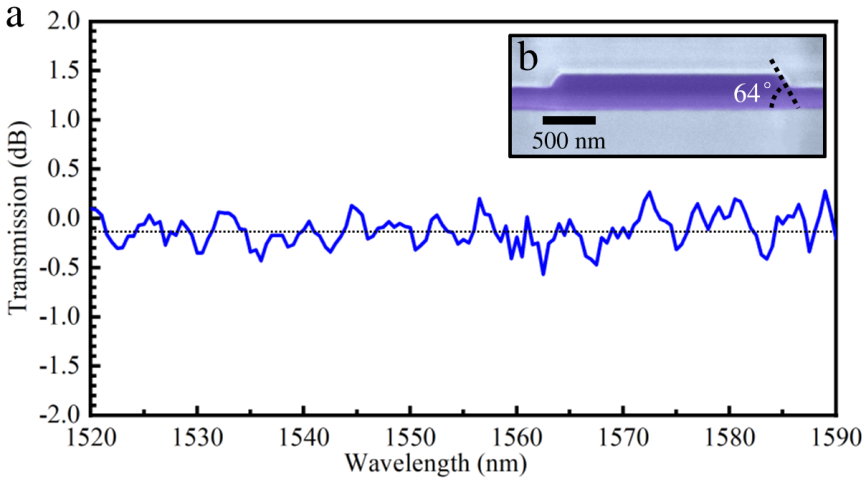


Fig. S3 **The propagation loss of the waveguide.** **a** The transmission of the 2 μm-width waveguides with a length of 1 cm. **b** The scanning electron image of the profile of the fabricated optical waveguide, showing a sidewall angle of 64°.

# III. The propagation losses of waveguide

Figure S3a shows that the propagation loss of the 2 μm-width waveguides is near 0.11 dB/cm. The scanning electron image of the profile of the optical waveguide presented in Fig. S3b indicates that the sidewall angle of the optical waveguide is equal to 64°.

# IV. The optimization of the gold electrodes

The profile of the electrodes is shown in Fig. S4a. The optical rid waveguides are located in the dielectric gaps between gold electrodes. The top width of the rid waveguide is 2 μm. The distance between the two electrodes is set at D_m_. For the voltage-length product (V_π_L), *D*_m_ should be as small as possible (red line of Fig. S4b). While a small D_m_ would result in a high additional optical Ohmic loss (blue line of Fig. S4b). The D_m_ set at 6 μm can well balance the trade-off.


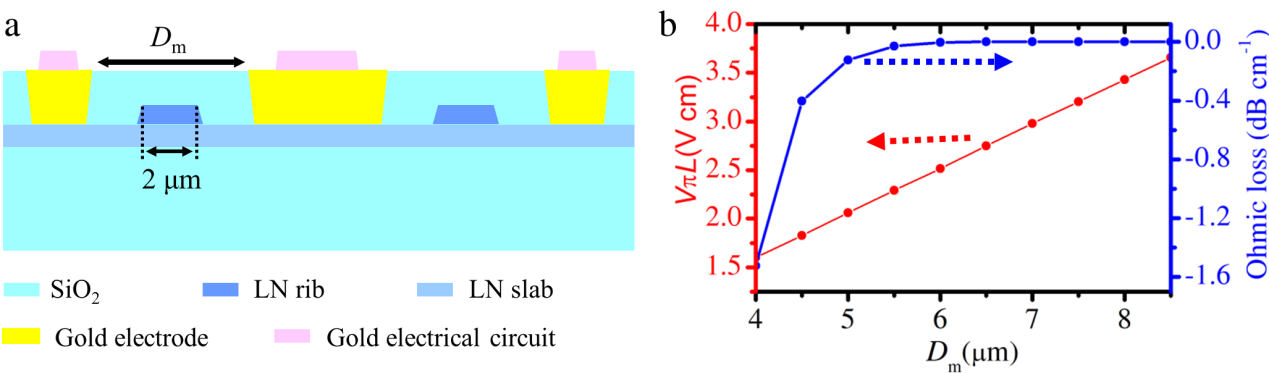


Fig. S4 **The design of the gold electrodes for the phase shifters.** **a** The profile of the phase shifters. **b** The simulation results of the voltage-length product and the Ohmic loss as the functions of the gap (*D*_m_) between two electrodes.

# V. The principle and experiment of the arbitrary polarization generation

**Principle**

Arbitrary SOP generation can be implemented using two PSRs, a Mach-Zehnder interferometer (MZI), as illustrated in Fig. S5a. Setting the input optical electrical field at $|\left. 1 \right\rangle$, the two components $|\left. H \right\rangle$ and $|\left. V \right\rangle$ of the output light can be given by

$\left( \begin{matrix} |\left. V \right\rangle\\ |\left. H \right\rangle\end{matrix} \right)=C_{\varphi}\times C_{M}\times C_{\theta}\times C_{M}\times\left( \begin{matrix} |\left. 1 \right\rangle\\ 0 \end{matrix} \right)$ (S1)

where *C*_M_, *C_θ_* and *C_φ_* are the transfer matrices of 3-dB 2×2 multimode interferometer (MMI) coupler, first and second electro-optic (EO) phase shifters, respectively. *C*_M_, *C_θ_* and *C*_φ_ can be expressed as:

$C_{M}=\frac{\sqrt{2}}{2}\left( \begin{matrix} 1 & e^{-i\frac{\pi}{2}} \\ e^{-i\frac{\pi}{2}} & 1 \end{matrix} \right)$, $C_{\theta}=\left( \begin{matrix} e^{i\frac{\theta}{2}} & 0 \\ 0 & e^{-i\frac{\theta}{2}} \end{matrix} \right)$, $C_{\varphi}=\left( \begin{matrix} e^{i\frac{\varphi}{2}} & 0 \\ 0 & e^{-i\frac{\varphi}{2}} \end{matrix} \right)$ (S2)

where *θ* and *φ* are the phase shifts of the two EO phase shifters, respectively. The relation between the $|\left. H \right\rangle$, $|\left. V \right\rangle$ and the output Stokes vector (*S*_0_, *S*_1_, *S*_2_, *S*_3_)^T^ can be given by,

$S_{0}=\left\langle H | H \right\rangle+\left\langle V | V \right\rangle$ (S3)

$S_{1}=\left\langle H | H \right\rangle-\left\langle V | V \right\rangle$ (S4)

$S_{2}=\left\langle V | H \right\rangle+\left\langle H | V \right\rangle$, (S5)

$S_{3}=i(\left\langle V | H \right\rangle-\left\langle H | V \right\rangle)$ (S6)

Based on Eqs. S1-S6, we can obtain that,

. (S7)

Therefore, controlling the voltages applied to the two EO phase shifters, we can generate an arbitrary pure polarization state. Fig. S5b-S5d presents the microscope images of the fabricated device.


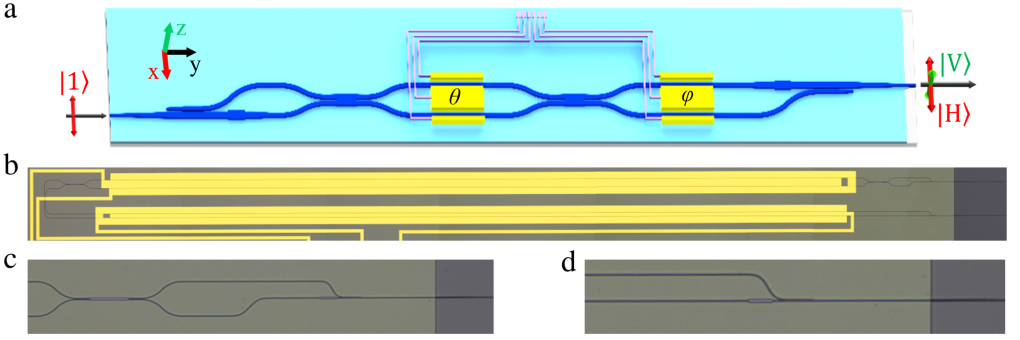


Fig. S5 **The model of the proposed polarization generating device.** **a** The schematic of the single-stage device. **b** The microscope image of the fabricated device. **c** and **d** are the enlarged images of the optical input and output ports, respectively.

In the above, we only consider the case of that the power splitting ratio of MMI coupler is equal to 50:50. However, in practice, the fabrication imperfections always cause the splitting ratio of MMI coupler to deviate from 50:50. To analyze this case, we set the power splitting ratio of MMI coupler at $\tau^{2}$:$\kappa^{2}$ (where $\tau^{2}+\kappa^{2}$ =1). The transfer matrix of MMI coupler can be updated as,

$C_{M^{'}}=\left( \begin{matrix} \tau& \kappa e^{-i\frac{\pi}{2}} \\ {\kappa e}^{-i\frac{\pi}{2}} & \tau\end{matrix} \right)$ (S8)

Based on Eqs. S1, S3-S5 and S8, we can obtain that

$S_{1}=4\kappa^{2}\tau^{2}\cos\theta-\left( \tau^{2}-\kappa^{2} \right)^{2}$ (S9)

Therefore, in this case, the maximum polarization extinction ratio (PER) near S_1_=1 can be given by,

$PER=-10\log_{10} \frac{1-{max(S}_{1})}{1+{max(S}_{1})}=-10\log_{10} \frac{\left( \tau^{2}-\kappa^{2} \right)^{2}}{4\kappa^{2}\tau^{2}}$ (S10)

Equation S10 shows that the PER is finite when $\tau$ is not equal to$\kappa$. To fully overcome this restriction, we further demonstrate a multi-stage device, in which additional interferometers function as beam splitters with variable splitting ratio (Fig. S6). In this case, we can obtain that,

$S_{1}=b^{2}\left\{ \sin\vartheta\sin\phi+a\left[ \cos\vartheta+\cos\vartheta\cos\phi+\cos\phi\right] \right\}-a^{3}$ (S11)

where $a=\tau^{2}-\kappa^{2}$, $b=2\kappa\tau$. $\vartheta$ and $\phi$ are the phase shifts of two phase shifters, respectively. $a\left[ \cos\vartheta+\cos\vartheta\cos\phi+\cos\phi\right]$ allows the multi-stage device to achieve a higher PER.


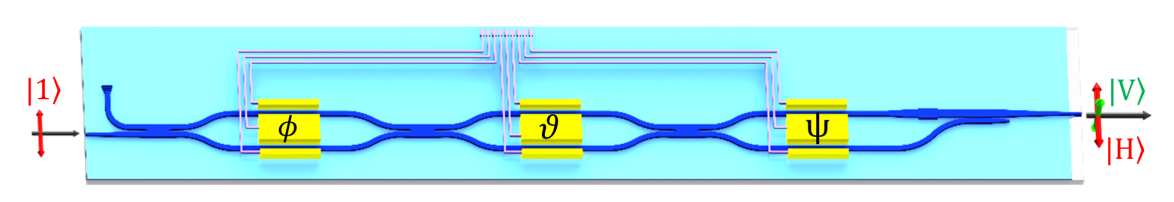


Fig. S6 The schematic of the multi-stage device. $\vartheta$, $\phi$ and $\psi$ are the phase shifts of three phase shifters, respectively.

**Experiment**

Fig. S7a presents the measured results of tuning a MZI circuit with EO phase shifter in 900 min, confirming the stability of EO tuning of the fabricated LN device. We can observe that the EO tuning still maintain stability after 22 days of the device was fabricated.


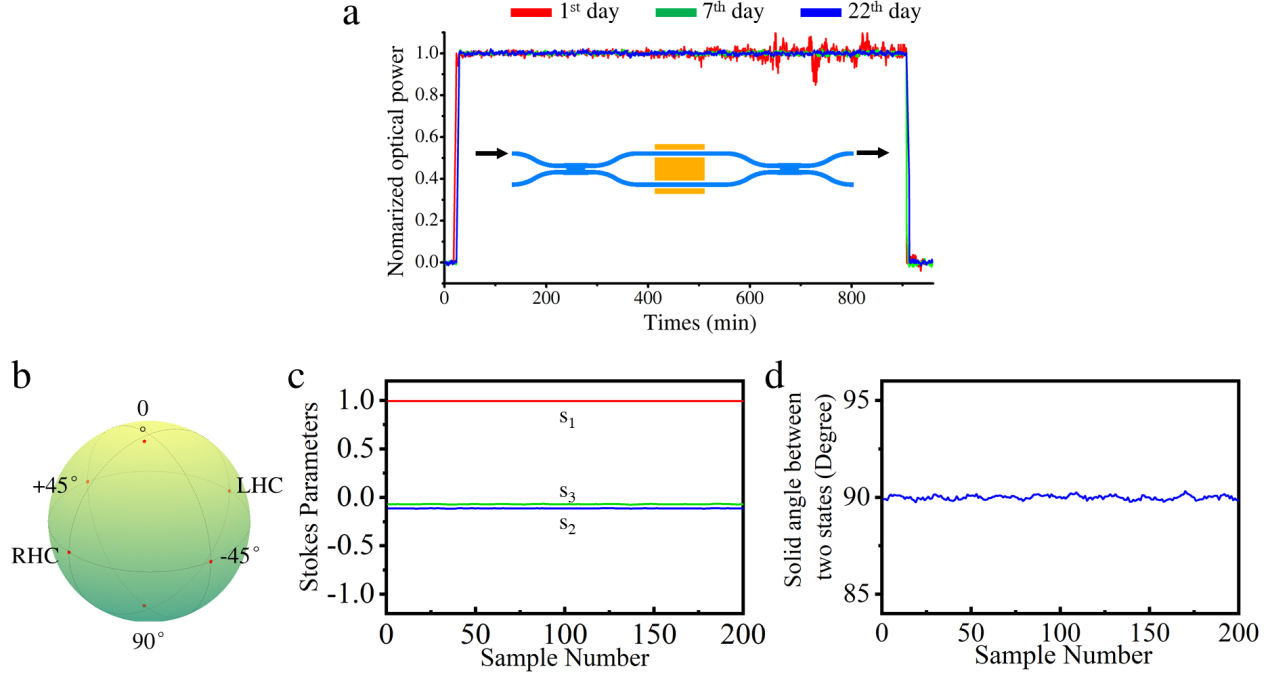


Fig. S7 **Repeatability measurement of our device by switching the generated SOPs among six states.** **a** The output power of a MZI circuit as a function of time after 1 day, 7 days and 22 days of the device was fabricated. **b** The six generated SOPs (six red dots) on a Poincaré sphere. **c** Stokes parameters of one of the generated SOPs as a function of sample points. **d** Solid angle between two switching states as a function of sample points.

Using a commercial polarization synthesizer/analyzer (General Photonics, PSY 201), we can characterize the performance of our device. Here, we would demonstrate that our device can generate highly repeatable polarization states at 0°, ±45°, 90° and LHC and RHC across a Poincaré sphere. Fig. S7b shows the repeatability of the six states on a Poincaré sphere by repeatedly switching among these states 200 times. We can observe that the six states (six red dots) are highly repeatable, with no discernible differences. It is worth noting that because the switching speed is so fast, the switching trajectory among different states cannot be captured by the commercial polarization synthesizer/analyzer. One of the generated SOPs presented in Fig. S7c indicates a high repeatability of the polarization generation. The other five generated SOPs also exhibit the same high repeatability. The solid angle between the two adjacent states is shown in Fig. S7d. The measured solid angle repeatability is 0.107°.

Activation loss measures the additional insertion loss caused in activating the device. It is defined as the difference of the maximum and minimum insertion losses of the device considering all possible activation conditions. The LNOI platform provides pure phase modulation which means the intensity of the light does not change with an external modulation voltage. This fundamentally avoids the activation loss commonly observed in silicon and InP devices. In fact, the measured activation loss in our device can be negligible (see Fig. S8).


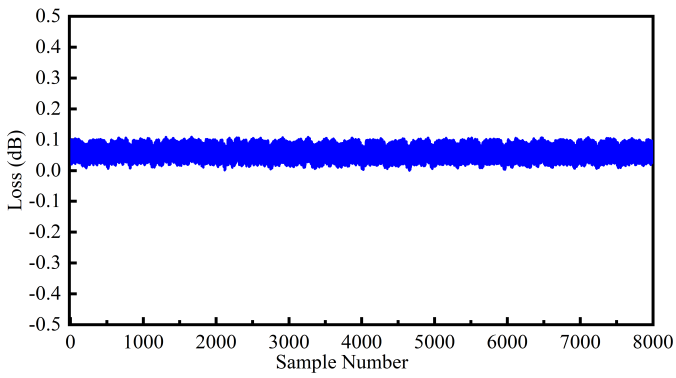


Fig. S8 The loss of our device when applying different voltages.

# VI. The principle and experiment of the polarization measurement

**Principle**

The proposed polarization measuring device is composed of a PSR, a MZI and a flip-chip bonded photodetector (PD), as depicted in Fig. S9a. It has an architecture similar to the SOP generating device, but with the PD for monitoring the optical intensity. The optical electrical field ($|\left. E_{PD} \right\rangle$) received by PD can be given by,

$|\left. E_{PD} \right\rangle=\left( \begin{matrix} 1 & 0 \end{matrix} \right)\times C_{M}\times C_{\theta}\times C_{M}\times C_{\varphi}\times\left( \begin{matrix} |\left. V \right\rangle\\ |\left. H \right\rangle\end{matrix} \right)$ (S12)

where $|\left. V \right\rangle$ and $|\left. H \right\rangle$ are two components of the input light. Based on Eqs. S2-S6 and S12, the relation between the optical intensity (*I*_PD_) and the input Stokes vector ($S_{0}^{'}$, $S_{1}^{'}$, $S_{2}^{'}$, $S_{3}^{'}$)^T^ can be given by,

$I_{\mathrm{PD}}=\left\langle E_{PD} | E_{PD} \right\rangle=\frac{1}{2}(S_{0}^{'}+S_{1}^{'}\cos\theta-S_{2}^{'}\sin\theta\cos\varphi+S_{3}^{'}\sin\theta\sin\varphi)$ (S13)

To reconstruct a full-Stokes vector, the proposed device needs to perform four optical intensity measurements (*I*_1_, *I*_2_, *I*_3_ and *I*_4_), which correspond to four drive voltage combinations of *θ* and *φ* phase shifters. According to Eq. S13, the input full-Stokes vector can be related to *I*_1_, *I*_2_, *I*_3_ and *I*_4_ by,

$\left( \begin{matrix} \begin{matrix} I_{1} \\ I_{2} \end{matrix} \\ \begin{matrix} I_{3} \\ I_{4} \end{matrix} \end{matrix} \right)=\mathbf{W}\times\left( \begin{matrix} \begin{matrix} S_{0}^{'} \\ S_{1}^{'} \end{matrix} \\ \begin{matrix} S_{2}^{'} \\ S_{3}^{'} \end{matrix} \end{matrix} \right)=\frac{1}{2}\left( \begin{matrix} \begin{matrix} 1 & \cos\theta_{1} \\ 1 & \cos\theta_{2} \end{matrix} & \begin{matrix} -sin\theta_{1}\cos\varphi_{1} & \sin\theta_{1}\sin\varphi_{1} \\ -sin\theta_{2}\cos\varphi_{2} & \sin\theta_{2}\sin\varphi_{2} \end{matrix} \\ \begin{matrix} 1 & \cos\theta_{3} \\ 1 & \cos\theta_{4} \end{matrix} & \begin{matrix} -sin\theta_{3}\cos\varphi_{3} & \sin\theta_{3}\sin\varphi_{3} \\ -sin\theta_{4}\cos\varphi_{4} & \sin\theta_{4}\sin\varphi_{4} \end{matrix} \end{matrix} \right)\times\left( \begin{matrix} \begin{matrix} S_{0}^{'} \\ S_{1}^{'} \end{matrix} \\ \begin{matrix} S_{2}^{'} \\ S_{3}^{'} \end{matrix} \end{matrix} \right)$ (S14)

where *θ*_i_ and *φ*_i_ are the phase shifts of two EO phase shifters when recording the i^th^ optical power, respectively. If the rank of the analysis matrix **W** is equal to 4, the proposed device can be used to reconstruct the full-Stokes vector. The reconstructed accuracy is mainly determined by the noise of the PD and the analysis matrix **W**. Comparing with the cost of decreasing the noise of the PD, the cost of optimizing the analysis matrix **W** can be ignored. For example, we only need to change the drive voltages of two phase shifters to optimize the analysis matrix. According to the previous theoretical analysis^1^, the analysis matrix **W** is optimal when the analysis matrix **W** has the properties of that:

$\mathbf{W}=\frac{1}{2}\left( \begin{matrix} \begin{matrix} 1 & 1/\sqrt{3} \\ 1 & -1/\sqrt{3} \end{matrix} & \begin{matrix} 1/\sqrt{3} & 1/\sqrt{3} \\ -1/\sqrt{3} & 1/\sqrt{3} \end{matrix} \\ \begin{matrix} 1 & 1/\sqrt{3} \\ 1 & -1/\sqrt{3} \end{matrix} & \begin{matrix} -1/\sqrt{3} & -1/\sqrt{3} \\ 1/\sqrt{3} & -1/\sqrt{3} \end{matrix} \end{matrix} \right)$ (S15)

or

$\mathbf{W}=\frac{1}{2}\left( \begin{matrix} \begin{matrix} 1 & -1/\sqrt{3} \\ 1 & 1/\sqrt{3} \end{matrix} & \begin{matrix} -1/\sqrt{3} & -1/\sqrt{3} \\ 1/\sqrt{3} & -1/\sqrt{3} \end{matrix} \\ \begin{matrix} 1 & -1/\sqrt{3} \\ 1 & 1/\sqrt{3} \end{matrix} & \begin{matrix} 1/\sqrt{3} & 1/\sqrt{3} \\ -1/\sqrt{3} & 1/\sqrt{3} \end{matrix} \end{matrix} \right)$ (S16)

To obtain the properties of Eqs. S15 and S16, $\theta_{i}$ (where *i* =1, 2, 3, and 4) can be equal to $\pm arccos(\frac{1}{\sqrt{3}})$ or $\pm[\pi-\arccos\left( \frac{1}{\sqrt{3}} \right)]$, and $\varphi_{i}$ can be equal to $\pm\pi/4$, $\pm3\pi/4$. *θ*_i_ and *φ*_i_ presented in Fig. S9b are two examples that allow us to obtain the optimal analysis matrix.


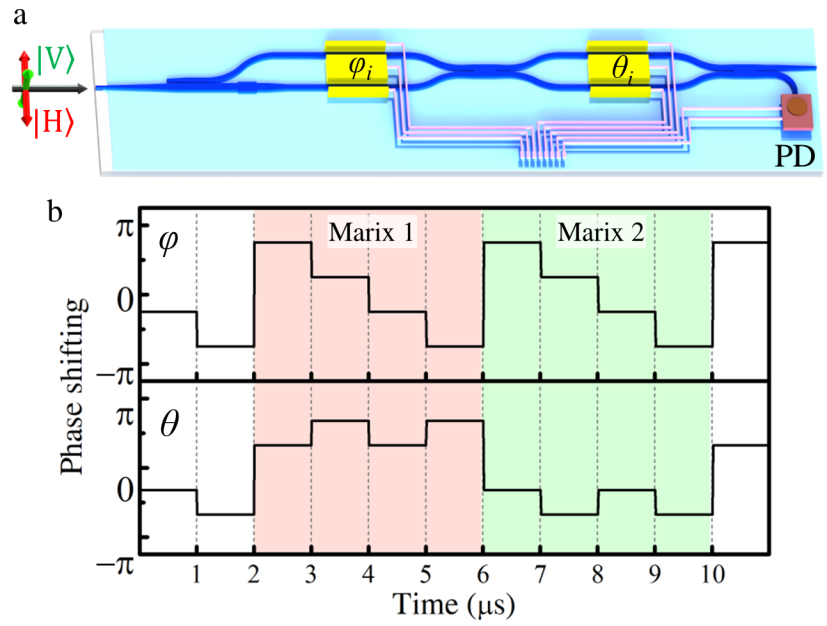


Fig. S9 **The model of the proposed polarization measuring device.** **a** The schematic of the proposed polarization measuring device. **b** The phase shifts of the *θ* and *φ* phase shifters when measuring the input polarization states.

The angle deviation between the input SOP and the measured one is used to characterize the performance of the polarization measurement, and can be given by,

$Angle deviation=\left| \cos^{-1} \left( \frac{\boldsymbol{S}_{\boldsymbol{m}}\boldsymbol{\cdot}\boldsymbol{S}_{\boldsymbol{in}}}{\left| \boldsymbol{S}_{\boldsymbol{m}} \right|*\left| \boldsymbol{S}_{\boldsymbol{in}} \right|} \right) \right|$ (S17)

where $\boldsymbol{S}_{\boldsymbol{m}}\boldsymbol{=}$($S_{m1}^{'}$, $S_{m2}^{'}$, $S_{m3}^{'}$)^T^ corresponds to the measured result, and $\boldsymbol{S}_{\boldsymbol{in}}\boldsymbol{=}$($S_{1}^{'}$, $S_{2}^{'}$, $S_{3}^{'}$)^T^ corresponds to the input SOP.

**Experiment**

Figure S10 presents the experiment setup for demonstrating our polarization measuring device. The light source consists of a tunable laser and an erbium-doped fiber amplifier (EDFA). Controlling the output optical power ratio between the tunable laser and EDFA, we can change the degree of polarization (DOP) of light source, because the light generated by EDFA is unpolarized and the light generated by the tunable laser is completely polarized. When measuring the results shown in Fig. 4 of the main text, we turned off the EDFA so that the light injected into the photonic chip is completely polarized. The input polarization states shown in Fig. 4 of the main text were randomly generated by a commercial polarization synthesizer/analyzer (General Photonics, PSY 201).

Next, we show that our device can also be used to measure the input light beam whose DOP is lower than 100%. In this case, the commercial polarization synthesizer/analyzer was used to measure the DOP of light which comes from the tunable laser and EDFA. Fig. S10b compares the DOP measured by our device and the commercial one, indicating that RMS deviation of the measured DOP from our device can achieve 2.5%.


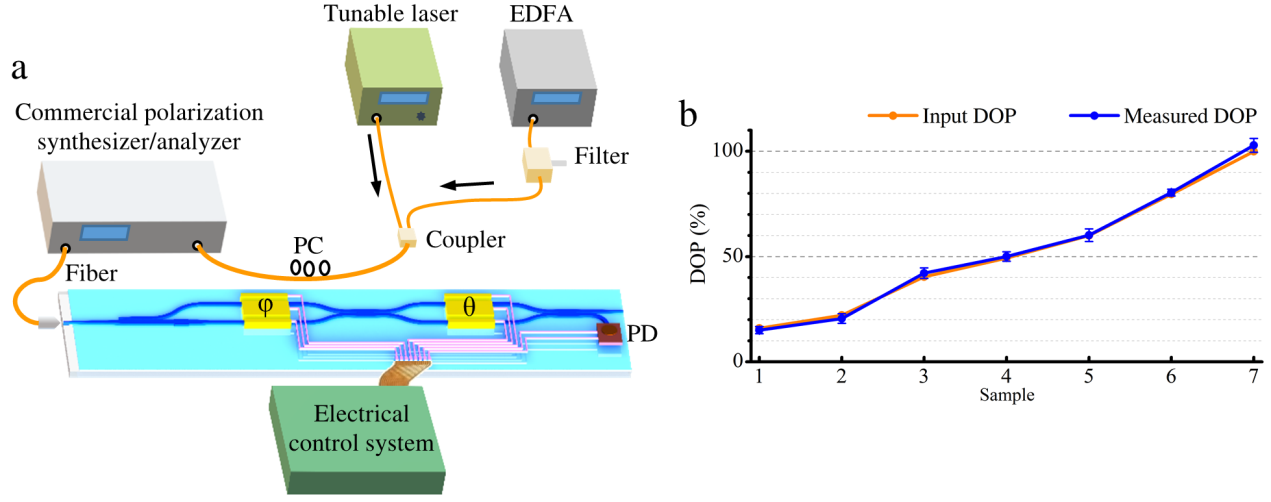


Fig. S10 **The experiment for demonstrating the proposed polarization measuring device. a** The schematic of experiment setup for demonstrating the proposed polarization measuring device. EDFA: erbium-doped fiber amplifier. PC: polarization controller. **b** The measured degree of polarization (DOP) from our device and the corresponding input DOP (orange line).

# VII. The experiment for polarization scrambling

The experimental setup for demonstrating polarization scrambling is presented in Fig. S11a. The light is emitted from a tunable laser, and passes through a polarization controller and then is injected into the proposed device by a lens fiber. Our device is controlled by a dual-channel arbitrary waveform generator. The arbitrary waveform generator can provide two triangle wave signals with different frequencies. The frequency ratio of the output signals was set at 1:$\sqrt{2}$. The scrambling rate can be controlled by the frequencies of two channels. According to Eq. S7, the scrambling rate can be calculated by:

$Scrambling rate=\sqrt{\left( \theta_{range}\times f_{\theta} \right)^{2}+\left( \varphi_{range}\times f_{\varphi} \right)^{2}}$ (S18)

where *θ*_range_ and *φ*_range_ are the phase shifting ranges of the *θ* and *φ* phase shifters, respectively. *f*_θ_ and *f*_φ_ are the frequencies of the triangle wave signals applied to *θ* and *φ* phase shifters, respectively. Here, we set *θ*_range_=4π and *φ*_range=_2π.

A commercial polarization analyzer (General Photonics, PSY 201) was used to characterize the generated polarization state. Fig. S11b provides the degree of polarization of the output light as a function of the scrambling rate of the fabricated polarization scrambling device when the detector integration time is set at 5 μs.


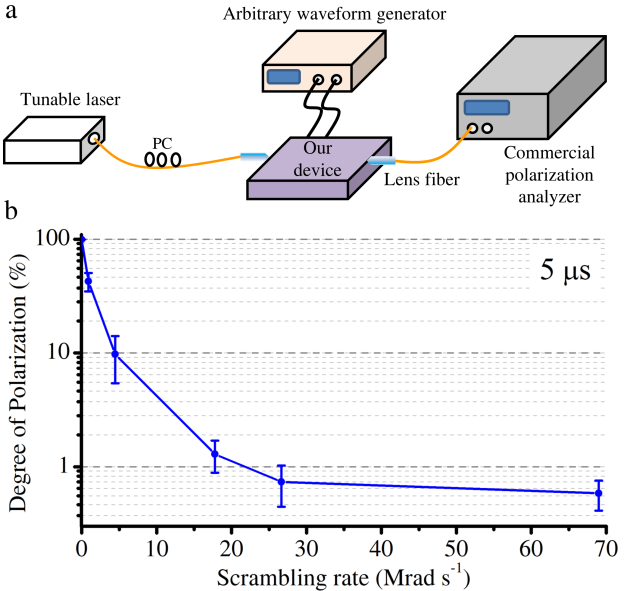


Fig. S11 **The experiment for polarization scrambling.** **a** The schematic of the experimental setup for demonstrating polarization scrambling. PC: fiber polarization controller. **b** The degree of polarization of the output light as a function of the scrambling rate of the fabricated device when the detector integration time is 5 μs.


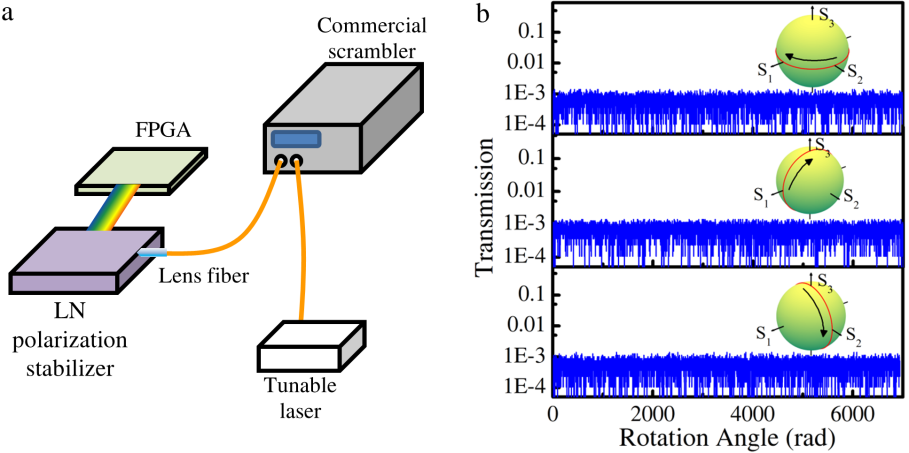


Fig. S12 **The experiment for demonstrating polarization control.** **a** The experiment setup for characterizing our device. **b** The transmission of the feedback signal as a function of the rotation angle of the input polarization. Insets: the trajectories of the input polarization states.

# VIII. The experiment for demonstrating polarization controlling device

The experiment setup of characterizing the endless automatic polarization controlling device is depicted in Fig. S12a. To quantify the control system, the feedback signal, including the deviations caused by the voltage modulation, is recorded every 5.1 μs. A commercial polarization scrambler (General Photonics, PSY 201) is used to scramble the polarization state. Using the commercial polarization scrambler, the trajectories of the input polarization state can rotate around a given axis of the Poincaré sphere. In the main manuscript, we show the results of using our device to track the input polarization state which rotates counterclockwise. Here, we show the results when the input polarization state rotates around the clockwise (Fig. S12b). It indicates that our device still works in these cases.

Although the architecture (i.e. single-stage device) with one PSR and one MZI which includes two EO phase shifters is sufficient for transforming any input SOP to the TE mode^2^, the reset process is inevitable when the driving voltages reach the boundaries (see Fig. S13), resulting in momentary SOP mismatch and data interruption. While the reset does not happen in the multi-stage device which includes four EO phase shifters (see Fig. S14).


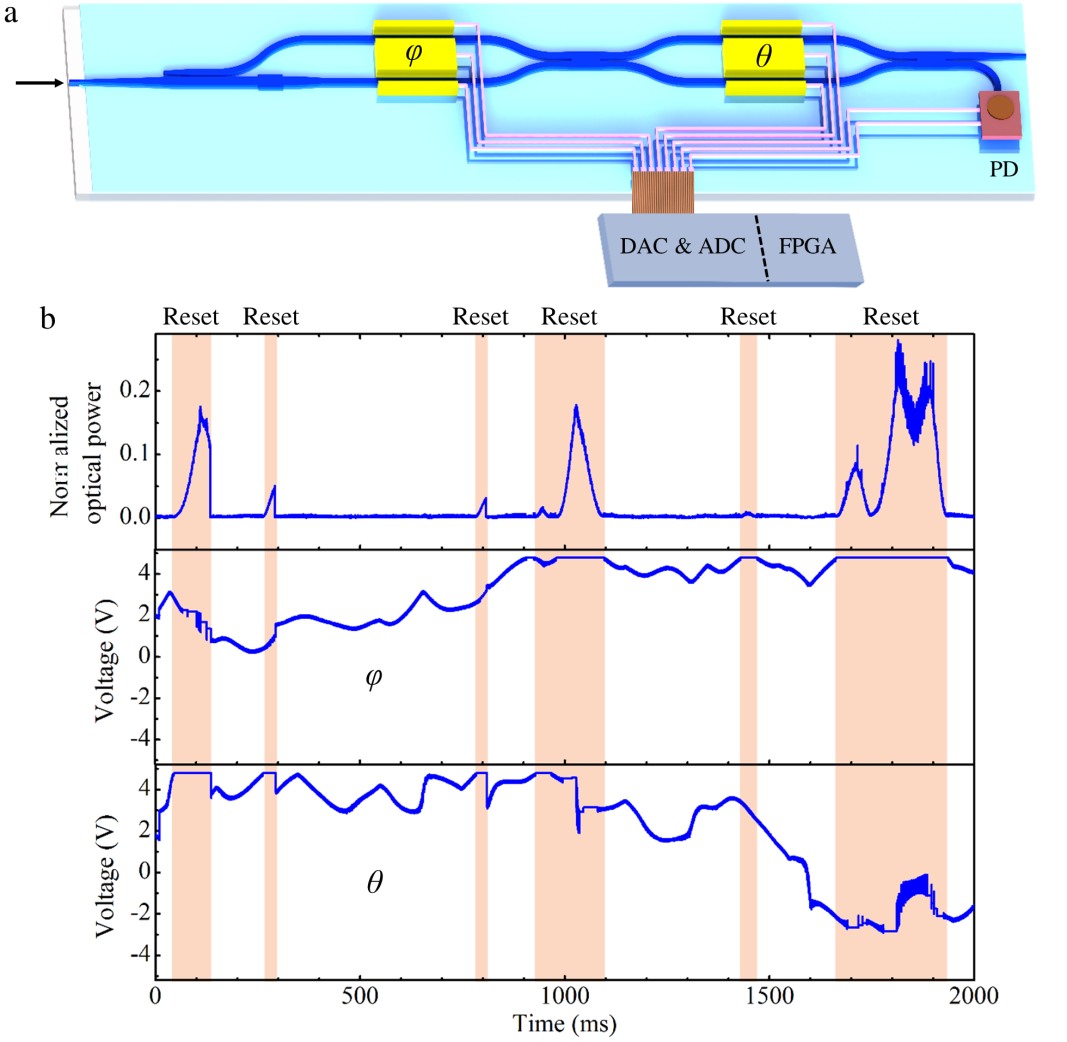


Fig. S13 **The experiment for characterizing single-stage polarization controlling device. a** The schematic of the single-stage polarization controlling device. **b** The normalized optical power of feedback signal, drive voltages of the *θ* and *φ* phase shifters as the functions of time.


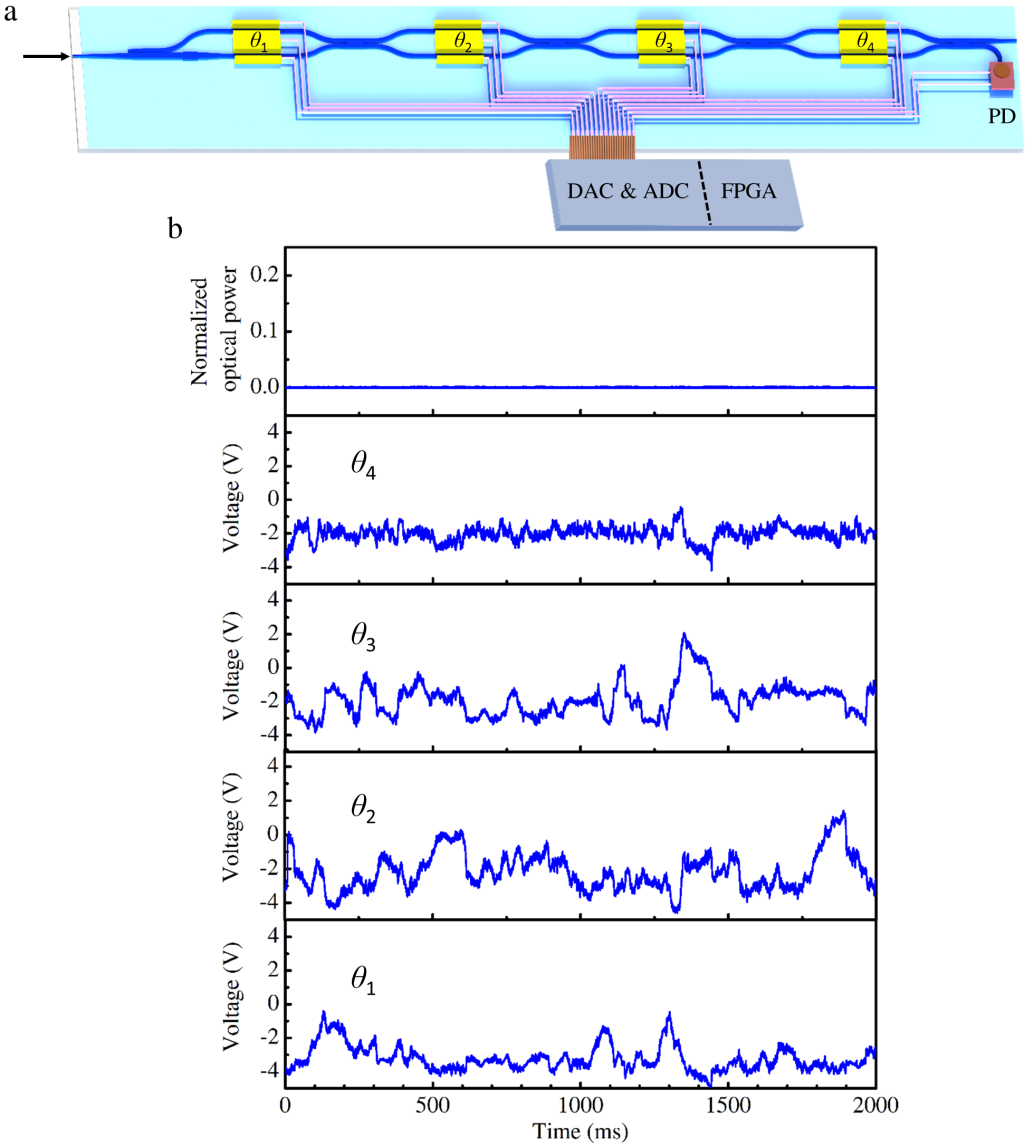


Fig. S14 **The experiment for characterizing multi-stage polarization controlling device. a** The schematic of the multi-stage polarization controlling device. **b** The normalized optical power of feedback signal, drive voltages of the corresponding four phase shifters as the functions of time.

# X. The summary of active bulky polarization management devices

Table 1 compares the performance of the active bulky polarization management devices. The active polarization management devices can be grouped into two kinds: the mechanical tuning device and the moving-part-free device. For the mechanical tuning device^3-5^, generally, the insertion loss is low, but the tuning speed is low and its stability needs to be improved. The fiber squeezed with the piezo-electric actuator can achieve a relatively high tuning speed, but its drive voltage and power consumption are high^5^.

The liquid crystal^6-9^, lanthanum zirconate titanate waveplate^10^, and magneto-optic crystal^11, 12^ are often applied by the bulky moving-part-free device to control the polarization states. They can achieve a relatively high tuning speed and are more repeatable. While they still require high drive voltage or high power consumption. For example, the lanthanum zirconate titanate waveplate can achieve a response time of 1 μs, but the V_π_ needs to be 200 V.

**Table 1** The summary of the active bulky polarization management devices.

| Material | Tuning method | Drive  Voltage  (V_π_) | | Power  for tuning | Speed  (or response time) |
| --- | --- | --- | --- | --- | --- |
| Birefringent waveplate^3^ | Mechanical rotation | - | - | | 960 rad/s |
| Fiber | Squeezing with piezo-electric actuator^5^ | 30V | ~20 W | | 752 krad/s |
|  | twist^4^ | - | - | | Low |
| Liquid crystal^7^ | Electro-optics | 40 V | - | | ~100 μs |
| Lanthanum zirconate titanate waveplate^10^ | Electro-optics | 200V | - | | ~1 μs |
| Magneto-optic  crystal^11, 13^ | Magneto-optics | < 5 V | 1.3 W | | ~150 μs |

# XI. Fabrication process

The proposed device was fabricated on X-cut LN-on-insulator (LNOI) wafer. The thicknesses of the LN and buried oxide layers are 360 nm and 4.7 µm, respectively. The fabrication process (Fig. S15) of the optical component is detailed in the following:

(i)-(ii) Electron beam lithography (EBL) was first used to define the rib waveguide structures on the hydrogen silses quioxane (HSQ) resist.

(iii) The patterns were transferred to the top LN layer with an etching depth of 180 nm by inductively coupled plasma (ICP) dry etching.

(iv)-(v) The strip waveguides were defined on the LN layer with an etching depth of 360 nm using EBL and ICP dry etching. The strip waveguide is used for the edge coupler.

(vi) A SiO_2_ layer with a thickness of 1 µm was deposited on the wafer as the upper-cladding by plasma-enhanced chemical vapor deposition (PECVD).


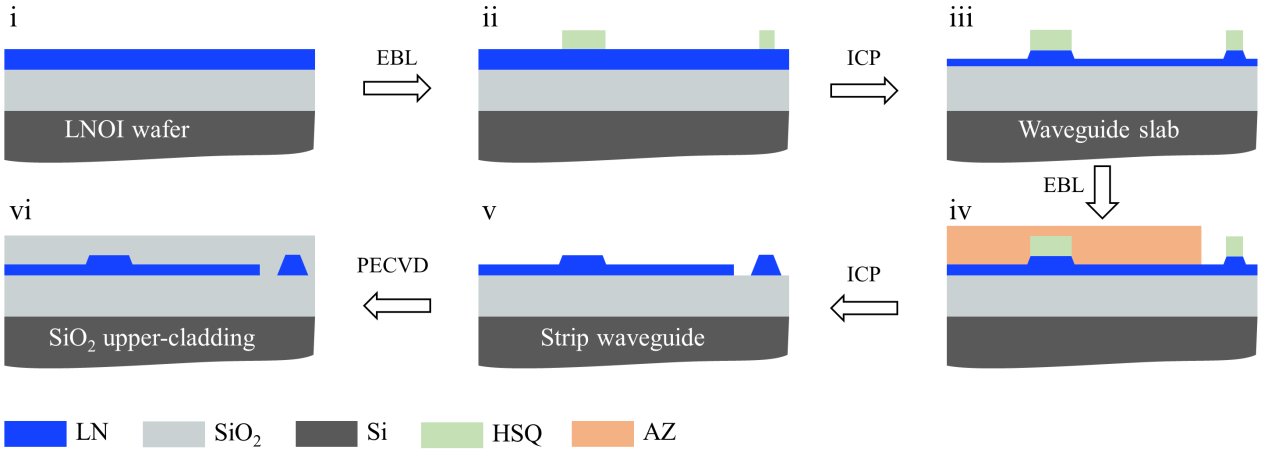


Fig. S15 **The schematic of the fabrication process of the optical components.** EBL: electron beam lithography; EBE: electron beam evaporation; ICP: inductively coupled plasma etching; PECVD: plasma-enhanced chemical vapor deposition. E-beam resists include HSQ, AZ.

# XII. Application in communication

**Experiment for mitigating the polarization-dependent gain of SOA**

To show an application in the communication, we combine our polarization controlling device with a semiconductor optical amplifier (SOA) to mitigate the polarization-dependent gain of SOA which can be used to improve the sensitivity of the optical receiver. Although lots of SOAs are polarization-independent, several polarization-dependent SOAs are also reported ^14^. However, the SOA with the assistance of the polarization controlling device can significantly mitigate its polarization dependence and attracts lots of attentions.


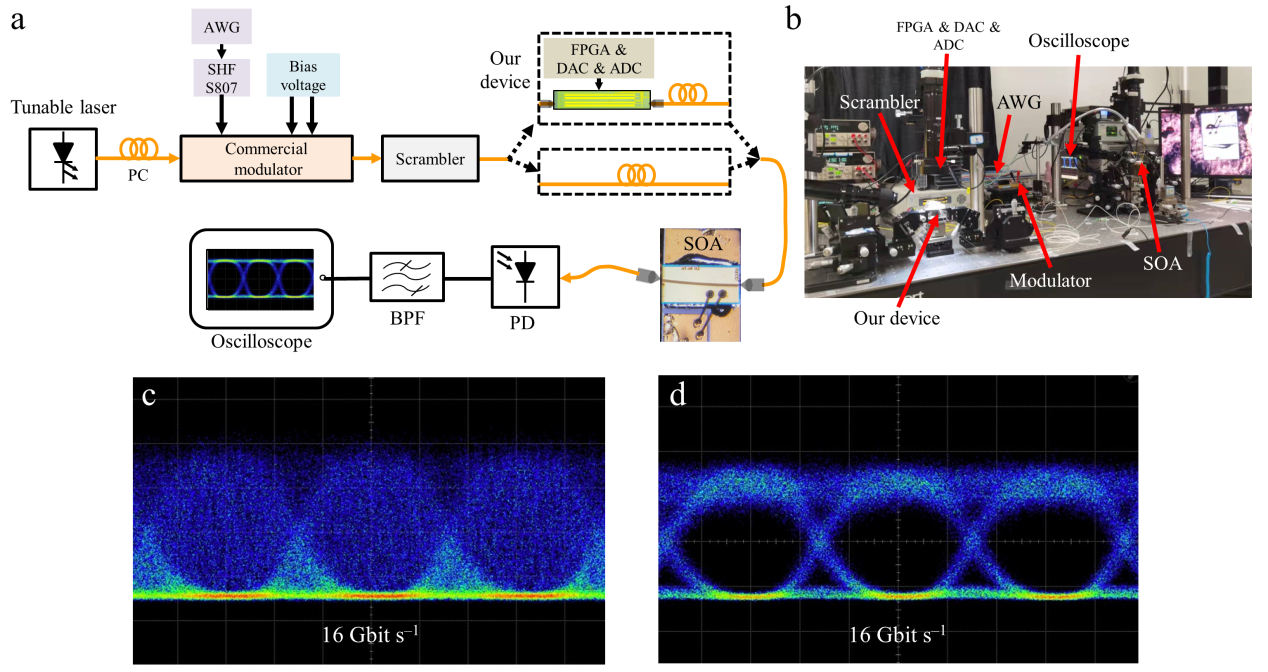


Fig. S16 **The experiment for demonstrating the application of the proposed polarization controlling device in the communication system.** **a** and **b** are the schematic and photograph of the experiment setup, respectively. PC: polarization controller based on the coiling fiber. AWG: arbitrary waveform generator. FPGA: field programmable gate array. SOA: semiconductor optical amplifier. PD: photodetector. BPF: band pass filter.

Fig. S16a and S16b present the schematic and photograph of the experiment setup, respectively. The light was emitted from a tunable laser (Santec, TSL-510), and modulated by a commercial electro-optic modulator (Thorlabs, LN05S-FC) at a transmission rate of 16 Gbit s^−1^. A commercial polarization scrambler (General Photonics, PSY 201) was used to scramble the polarization state of the light. In the experiment, we set the scrambling rate at 6.28 Krad/s. Then, two paths could be selected for comparing the performances of the systems with and without our device. One path includes our on-chip endless automatic polarization controlling device, and the other one excludes that. Before the light injected into the photodetector, the light was amplified by an on-chip commercial SOA (Thorlabs, BOA1007C). An oscilloscope (Agilent, DCA-X 86100D) was used to characterize the quality of the received data.

Here, we have demonstrated that the on-chip endless automatic polarization controlling device can well improve the polarization dependence of the on-chip SOA. Moreover, the polarization control is also a powerful operation to improve the performance of the coherent communication system.^15^ Recently, polarization control has re-emerged as a research hot topic due to the demand for low-cost coherence for future data-center interconnections (DCIs). According to the Ethernet roadmap, 800 Gb/s and 1.6 Tb/s will be the next-generation DCI interface speeds after 2023. In this scenario, coherent technology becomes a promising and competitive option due to higher sensitivity and stronger tolerance to optical impairments compared to intensity modulation/direct detection (IM/DD). But the expensive cost and high power consumption are still major issues, due to the demand for narrow-linewidth lasers and complex digital signal processing (DSP) algorithms. Thus, it is very important to reduce the cost and power consumption for coherent technology. This is where the polarization control comes in, whereby a polarization controller can be co-integrated at the receiver end of the coherent receiver to greatly simplify the DSP algorithm and reduce power-consumption. For example, using an automatic polarization control with a polarization state tracking speed of 400 rad s^-1^, a coherent communication system only require a low-cost DFB laser with 1MHz linewidth to support ~2km 800-Gb s^-1^ real-time transmission with multiple input multiple output-free DSP ^16^.

There are some polarization rotation problems in optical communication applications that cannot be handled by DSP alone. For example, lightening can cause polarization rotations at speeds an order of magnitude higher than typical, which may be faster than the DSP’s ability to maintain tracking. In fact, there is evidence that the DSP often fails to track the changes of polarization state and polarization-mode dispersion caused by lightning strike for optical transmission in aerial fiber ^17^. Note that 50% of all direct lightning strikes could generate a polarization state change of larger than 400 krad s^-1^, and meanwhile, the top 1% of strongest lightning strikes might generate a rotational speed even higher than 2 Mrad s^-1^ ^17^. An ultra-high speed polarization controlling device is well suited to address this issue. We believe our device have the potential to capture even the fastest lightning-induced events.

**The potential application of our device in self-homodyne coherent detection**

Traditional coherent detection used in metro and long-haul networks, with higher receiver sensitivity and spectral efficiency, provides a scalable solution for short-reach systems in data center. Nonetheless, it is still too expensive with high power consumption, due to the demand of narrow-linewidth lasers and complex digital signal processing (DSP) algorithms. Consequently, the self-homodyne coherent detection (SHCD) has recently been proposed for data center applications. The main concept of SHCD system is to send the modulated signal and a copy of tone as local oscillator (LO) originating by the same laser from transmitter (TX) to receiver (RX) for coherent reception. It can minimize the impact of laser phase noise and omit the frequency offset, thus allows the use of un-cooled lasers with large linewidth and simplifies the DSP algorithms ^18-20^. However, polarization management is indispensable to compensate the polarization fluctuation of the received LO. To further reduce the DSP power consumption and keep the spectral efficiency (SE) of standard coherent system, the bidirectional (BiDi) SHCD system with automatic polarization controller has been proposed to combat the polarization fading of remote LO ^21^.

To date, most of the polarization controlling devices in SHCD system are based on silicon photonic devices with thermal phase shifter ^15^, which are with slow response speed and large power consumption. The LNOI-based endless automatic polarization controllers demonstrated here offers a very promising solution, because of the high speed, low-insertion loss and low power consumption. In ref. [15], a silicon photonic LO polarization tracking device with tracking speed of 300 rad/s has been used. In contrast, our device shows a better endless polarization tracking speed of >10K rad/s. Moreover, high speed PD and hybrids can be further integrated with our devices to offer and monolithically integrated solution for SHCD, as depicted in Fig. S17.


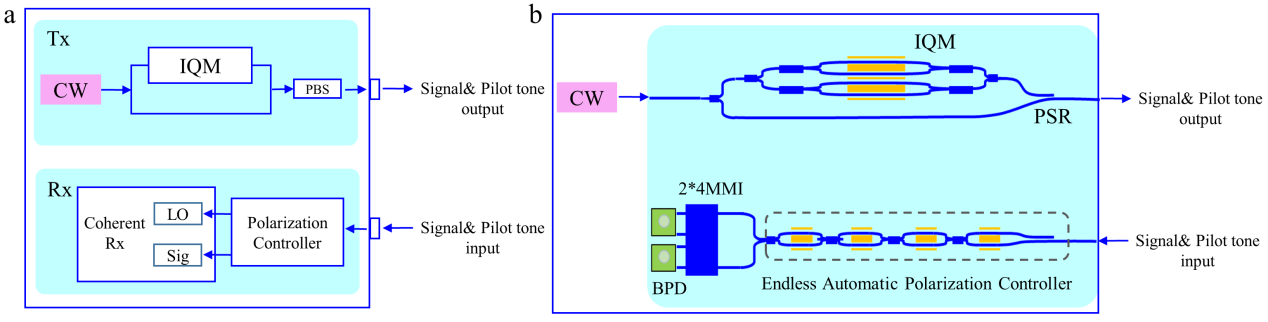


Fig. S17 **a** Architecture of the SHCD. **b** The proposed monolithically integrated solution for SHCD based on LNOI. IQM: IQ modulator; PBS: polarization beam splitter; PC: polarization controller; mBPD: monitoring balance photodetector; BPD: balance photodetector. PSR: polarization splitter-rotator.

# XIII. Potential applications in the sensing fields

The polarization management devices demonstrated in this works include polarization measurement device, polarization generation device, polarization scrambling device, and polarization tracking device. These devices can realize the most of the essential functionalities related to the polarization state, and have a huge potential application in sensing fields, such as fiber optic current sensing ^22, 23^, seismic and water wave sensing ^24^, body joint motion sensing ^25^, polarization light remote detection and ranging (LiDAR), and Muller matrix polarimetry system. Fig. S18 summarizes some potential sensing applications.

**Fiber optic current sensing system** (Fig. S18a). When a fiber passes through a magnetic field induced by an electrical current, its output SOP linearly varies with the value of the current due to the Faraday effect ^22^. Therefore, the value of a current can be obtained by measuring the SOP of light. This method has a minimal electrical interference on the signal line. Our devices offer a compact, fast and low-cost polarization measurement solution.

**Eismic and water wave sensing system** (Fig. S18b). In 2021, Zhan et al. reported an optical polarization–based seismic and water wave sensing on transoceanic cable ^24^. They successfully sensed seismic and water waves over an ultra-long submarine cable connecting several cities in America, by monitoring the polarization of regular optical telecommunication channels. This method has the potential to convert global submarine cables into real-time earthquake and tsunami sensing systems. Our devices offer a compact and real-time polarization measurement solution.

**Body joint motion sensing system** (Fig. S18c). In 2007, Donno et al. reported a body joint motion sensing system based on a single-mode fiber and a polarization measurement device ^25, 26^. They successfully sensed the joint angle of flexion and extension with a range of the relative angle of 90◦ and a resolution of 0.01◦. This method has the advantages of durable, flexible, and low-cost. Our devices offer a compact and fast polarization measurement solution.

**Polarization measurement-based optical spectrum analyzer** (Fig. S18d). In 2008, Yao et al. reported an optical spectrum analyzer using a polarization measurement device and a differential group delay (DGD) module ^27^. They successfully measured the spectrum of swept laser sources at a repetition rate of more than 100 kHz. Our devices offer a compact and fast polarization measurement solution.

**Distributed transverse-force sensing system** (Fig. S18e). In 2020, Feng et al. reported a direct distributed transverse-force sensing system based on a single-mode fiber, a polarization generation device, and a polarization measurement device ^28^. Their method features a minimum detectable transverse line-force of 6.61×10^-4^ N/mm, a spatial resolution of 3.7 mm, and a sensing distance of 103.5 m. Our devices offer a compact, fast, and high-accuracy polarization generation and measurement solution.


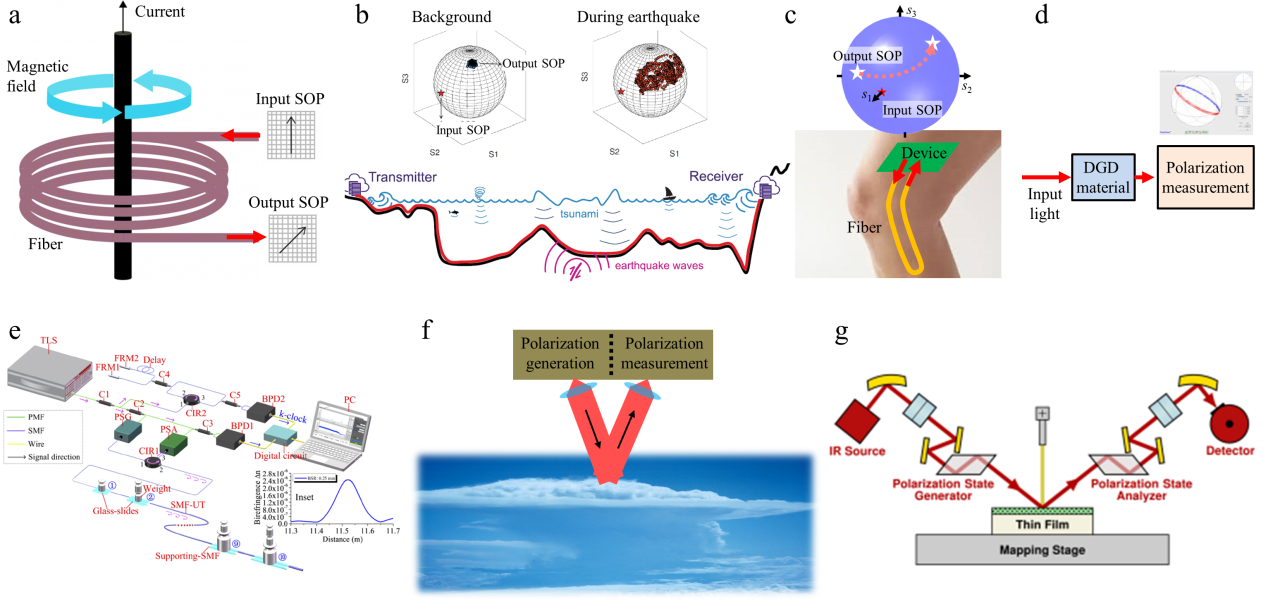


Fig. S18 **Some potential applications of the proposed devices.** **a**-**g** are the schematics of applications in fiber optic current sensing (reproduced from [23]), seismic and water wave sensing (reproduced from [24]), body joint motion sensing, polarization measurement-based optical spectrum analyzer (reproduced from [27]), distributed transverse-force sensing (reproduced from [28]), polarization LiDAR, and Muller matrix polarimetry system (reproduced from [29]), respectively.

**Polarization LiDAR system** (Fig. S18f). By controlling the SOP of the emitted light and analyzing the SOP of the reflected signal, a polarization LiDAR system can reveal the profiles and types of aerosol particles, which is important for monitoring air pollution and predicting climate change ^30, 31^. Our devices offer a compact, fast, and high-accuracy polarization generation and measurement solution, and can significantly improve the throughput and spatial-temporal resolutions.

**Muller matrix polarimetry system** (Fig. S18g). A Mueller matrix contains rich microstructural information about samples, and it can be obtained by simultaneously performing polarization generation and measurement. The Muller matrix polarimetry system can provide essential sensing, diagnostic, analytical, and metrology tools in the fields of material analysis, chemical, pharmaceutical, biomedical, metal, and semiconductor integrated-circuit (IC) industries ^29, 32^. Our devices offer a compact and fast polarization generation and measurement solution.

# XIV. Body joint motion sensor

Here, we demonstrate that the proposed polarization measurement device can be used to build a body joint motion sensor. The principle of the proposed sensor is that the output SOP of a single-mode fiber varies with changes in the birefringence of the fiber due to elasto-optic effect when twisting or bending the fiber. When a single-mode fiber is twisted, the elasto-optic effect leads to the appearance of circular birefringence, and thus the linear polarization can be preserved but the polarization plane will be rotated along the propagated direction of light as shown in Fig. S19a ^33^. When a single-mode fiber is bent, the distribution of stress is asymmetrical at x and y directions as shown in S19b, and thus the variation of the effective index at x and y directions due to the elasto-optic effect is different, resulting in the appearance of linear birefringence ^34^.

Fig. S19c presents the experiment setup for demonstrating the body joint sensor. Here, a robot arm was used to simulate the body joint movement, such as pronation/supination and flexion/extension. A fiber was fixed along the robot arm and passed through the joints of the robot arm. The output SOP from the fiber was monitored by our polarization measurement device.

We used two joints in the experiment: one that allows pronation and supination, and the other that allows flexion and extension. The rotation range and speed of joint angle were programmed to be 54 degrees and 315 degree s^-1^, respectively. For each measurement, we recorded 8 cycles. One cycle includes the motions: 1) the robot arm performs supination (or flexion) from 27˚ to -27˚; 2) Stopping movement for 200 ms; 3) the robot arm performs pronation (or extension) from -27˚ to 27˚; 4) Stopping movement for 200 ms.

Fig. S20a shows the measured Stokes parameters vary with time as the robot arm performs pronation and supination movement. After calibrating the relation between the Stokes parameter and the joint angle of pronation/supination, we can obtain information on the joint angle at each moment, as shown in Figure S20b. The response speed of the sensor can be very fast thanks to the fast speed from the polarization measurement device.

We used the same method to sense the joint angle when the robot arm performs the flexion/extension movement, and the results are depicted in Fig. S21.


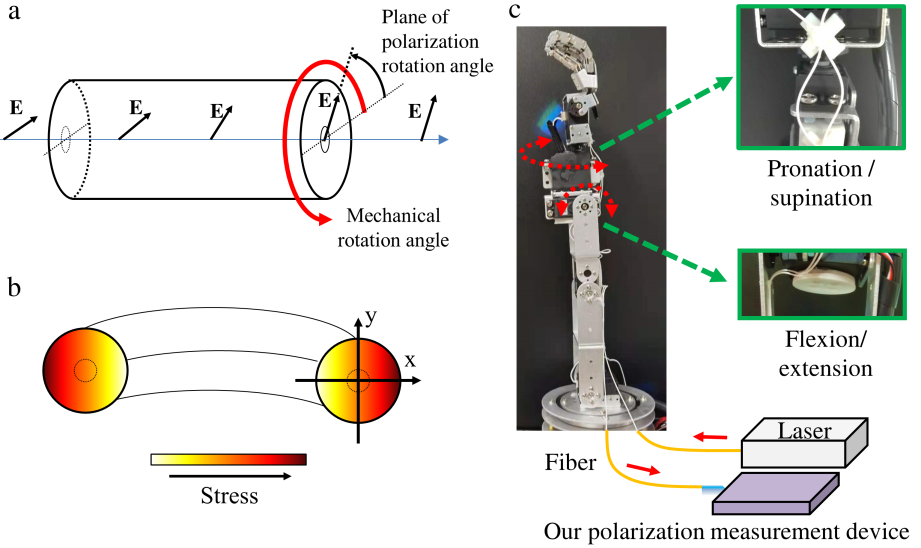


Fig. S19 **a** The principle of polarization rotation due to twisting. **b** The stress distribution when the fiber is bent. **c** The experiment setup for demonstrating body joint motion sensor.


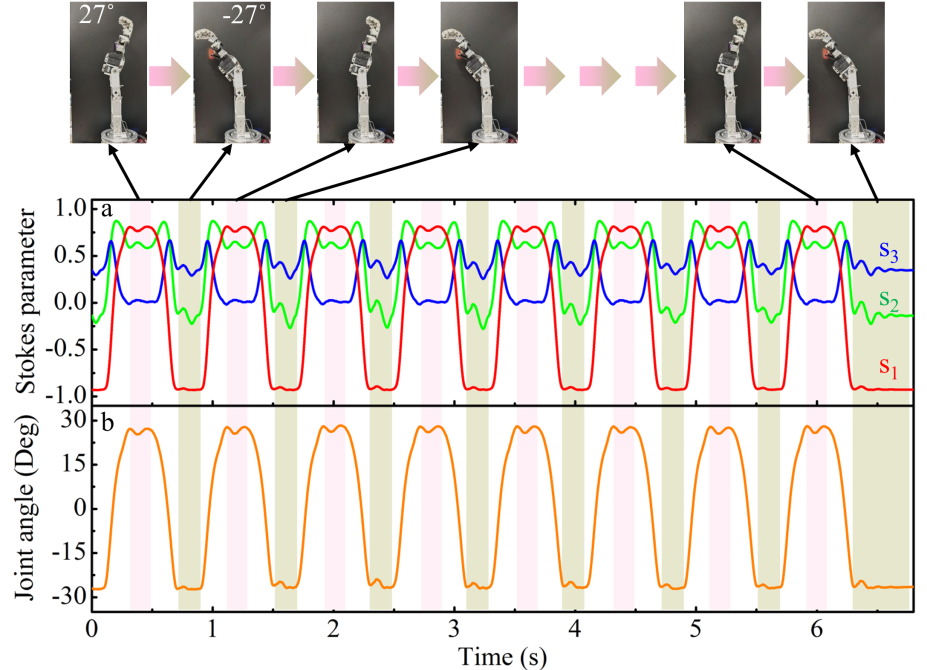


Fig. S20 **The experiment for sensing the motions of pronation and supination.** **a** and **b** are the measured Stokes parameters and reconstructed joint angle vary with time, respectively, when the robot arm performs the motions of pronation and supination. Top: the situation of robot arm at each moment. Blue arrow lines mark the direction of the hand of the robot arm. Color areas (pink and gray) mark the period of the robot arm is stopping movement.


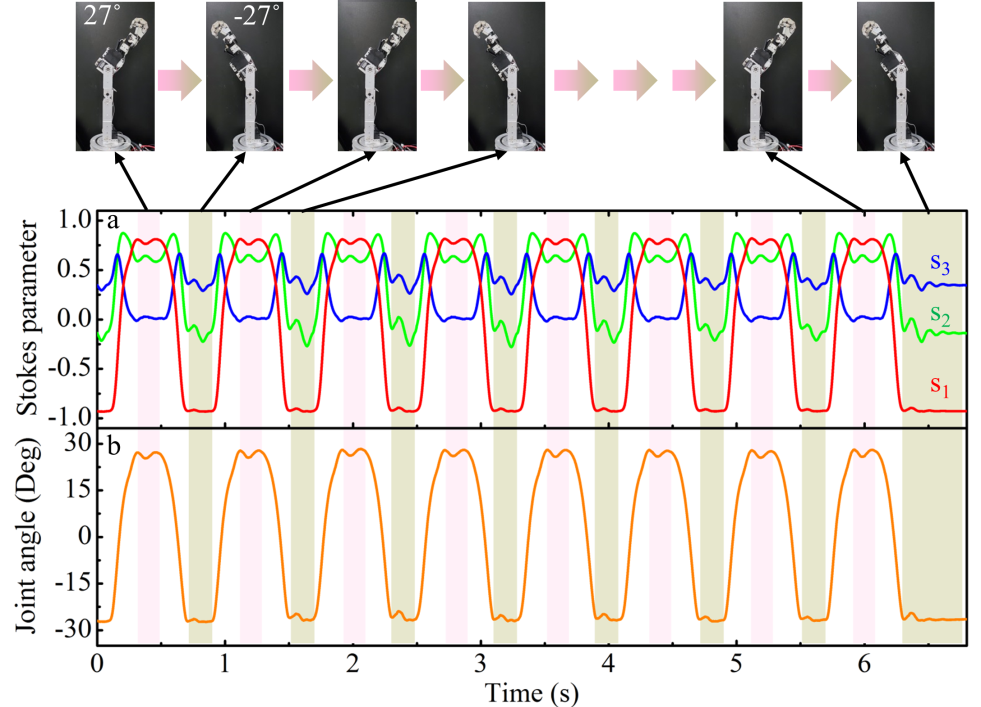


Fig. S21 **The experiment for sensing the motions of flexion and extension.** **a** and **b** are the measured Stokes parameters and reconstructed joint angle vary with time, respectively, when the robot arm performs the motions of flexion and extension. Top: the situation of robot arm at each moment. Color areas (pink and gray) mark the period of the robot arm is stopping movement.

# Reference

1. Goudail, F. Noise minimization and equalization for Stokes polarimeters in the presence of signal-dependent Poisson shot noise. *Optics letters* **34**, 647-649 (2009).

2. Ma, M. et al. Automated control algorithms for silicon photonic polarization receiver. *Optics express* **28**, 1885-1896 (2020).

3. Bobach, S., Hidic, A., Arlt, J.J. & Hilliard, A.J. Note: A portable rotating waveplate polarimeter. *The Review of scientific instruments* **88**, 036101 (2017).

4. Smith, A.M. Birefringence induced by bends and twists in single-mode optical fiber. *Appl Opt* **19**, 2606-2611 (1980).

5. Yao, L., Huang, H., Chen, J., Tan, E. & Willner, A. A novel scheme for achieving quasi-uniform rate polarization scrambling at 752 krad/s. *Optics express* **20**, 1691-1699 (2012).

6. Zhuang, Z.Z., Suh, S.W. & Patel, J.S. Polarization controller using nematic liquid crystals. *Optics letters* **24**, 694-696 (1999).

7. Dupont, L., Sansoni, T. & de la Tocnaye, J.L.D. Endless smectic A* liquid crystal polarization controller. *Optics Communications* **209**, 101-106 (2002).

8. Pitilakis, A.K., Zografopoulos, D.C. & Kriezis, E.E. In-Line Polarization Controller Based on Liquid-Crystal Photonic Crystal Fibers. *Journal of Lightwave Technology* **29**, 2560-2569 (2011).

9. Chiba, T., Ohtera, Y. & Kawakami, S. Polarization stabilizer using liquid crystal rotatable waveplates. *Journal of Lightwave Technology* **17**, 885-890 (1999).

10. Bourderionnet, J. et al. Endless Fiber-to-Fiber Polarization Controller Based on Ceramic Programmable Waveplates. *IEEE Photonics Technology Letters* **16**, 1101-1103 (2004).

11. Goldring, D., Zalevsky, Z., Shabtay, G., Abraham, D. & Mendlovic, D. Magneto-optic-based devices for polarization control. *J Opt a-Pure Appl Op* **6**, 98-105 (2004).

12. Stoyanova, E., Ivanov, S. & Rangelov, A. Arbitrary polarization control by magnetic field variation. *Appl Opt* **59**, 10224-10227 (2020).

13. Zhang, Y. et al. Complete polarization controller based on magneto-optic crystals and fixed quarter wave plates. *Optics express* **14**, 3484-3490 (2006).

14. Op de Beeck, C. et al. III/V-on-lithium niobate amplifiers and lasers. *Optica* **8**, 1288 (2021).

15. Gui, T. et al. Real-Time Demonstration of 600 Gb/s DP-64QAM Self-Homodyne Coherent Bi-Direction Transmission with Un-Cooled DFB Laser. *2020 Optical Fiber Communications Conference and Exposition (Ofc)* (2020).

16. Wang, L. et al. First Real-time MIMO-free 800Gb/s DP-64QAM Demonstration Using Bi-Directional Self-homodyne Coherent Transceivers. *ECOC* (2021).

17. LIGHTWAVE (2016).

18. Cheng, J. et al. Comparison of Coherent and IMDD Transceivers for Intra Datacenter Optical Interconnects. *OFC* (2019).

19. Morsy-Osman, M. et al. DSP-free 'coherent-lite' transceiver for next generation single wavelength optical intra-datacenter interconnects. *Optics express* **26**, 8890-8903 (2018).

20. Sowailem, M.Y.S. et al. Self-homodyne system for next generation intra-datacenter optical interconnects. *Optics express* **25**, 27834-27844 (2017).

21. Gui, T. et al. Real-Time Demonstration of Homodyne Coherent Bidirectional Transmission for Next-Generation Data Center Interconnects. *Journal of Lightwave Technology* **39**, 1231-1238 (2021).

22. Silva, R.M. et al. Optical Current Sensors for High Power Systems: A Review. *Applied Sciences* **2**, 602-628 (2012).

23. <https://fibercore.humaneticsgroup.com/perspectives/2020/07/17/fiber-optic-current-sensors-and-optical-current-transformers>.

24. Zhan, Z. et al. Optical polarization–based seismic and water wave sensing on transoceanic cables. *Science* **371**, 931-936 (2021).

25. Faisal, A.I. et al. Monitoring Methods of Human Body Joints: State-of-the-Art and Research Challenges. *Sensors* **19** (2019).

26. Donno, M., Palange, E., Di Nicola, F., Bucci, G. & Ciancetta, F. A New Flexible Optical Fiber Goniometer for Dynamic Angular Measurements: Application to Human Joint Movement Monitoring. *IEEE Transactions on Instrumentation and Measurement* **57**, 1614-1620 (2008).

27. Yao, X.S., Zhang, B., Chen, X. & Willner, A.E. Real-time optical spectrum analysis of a light source using a polarimeter. *Optics express* **16**, 17854-17863 (2008).

28. Feng, T., Zhou, J., Shang, Y., Chen, X. & Steve Yao, X. Distributed transverse-force sensing along a single-mode fiber using polarization-analyzing OFDR. *Optics express* **28**, 31253-31271 (2020).

29. Furchner, A. et al. Ultrasensitive broadband infrared 4 × 4 Mueller-matrix ellipsometry for studies of depolarizing and anisotropic thin films. *Journal of Vacuum Science & Technology B* **38**, 014003 (2020).

30. Shaw, J.A., Sassen, K. & Tyo, J.S. Polarization in lidar: a review. **5158**, 151 (2003).

31. Nicolae, D. et al. Polarization lidar for atmospheric monitoring. *EPJ Web of Conferences* **176**, 05052 (2018).

32. He, H. et al. Mueller Matrix Polarimetry—An Emerging New Tool for Characterizing the Microstructural Feature of Complex Biological Specimen. *Journal of Lightwave Technology* **37**, 2534-2548 (2019).

33. Budinski, V. & Donlagic, D. Fiber-Optic Sensors for Measurements of Torsion, Twist and Rotation: A Review. *Sensors* **17** (2017).

34. Ulrich, R., Rashleigh, S.C. & Eickhoff, W. Bending-induced birefringence in single-mode fibers. *Optics letters* **5**, 3 (1980).
